# Inhibition of BAD-Ser99 phosphorylation synergizes with PARP inhibition to ablate *PTEN*-deficient endometrial carcinoma

**DOI:** 10.1038/s41419-022-04982-8

**Published:** 2022-06-20

**Authors:** Xi Zhang, Peng Huang, Liqiong Wang, Shu Chen, Basappa Basappa, Tao Zhu, Peter E. Lobie, Vijay Pandey

**Affiliations:** 1 Shenzhen Bay Laboratory, Shenzhen, 518055 Guangdong China; 2 Tsinghua Berkeley Shenzhen Institute, Tsinghua Shenzhen International Graduate School, Tsinghua University, Shenzhen, 518055 PR China; 3 Department of Gynecology and Obstetrics, the University of Hong Kong-Shenzhen Hospital, Shenzhen, 518053 Guangdong, China; 4 Department of Studies in Organic Chemistry, University of Mysore, Manasagangotri, 570006 Mysore India; 5 Department of Oncology of the First Affiliated Hospital, Division of Life Sciences and Medicine, University of Science and Technology of China, Hefei, Anhui 230027 China; 6 Hefei National Laboratory for Physical Sciences, the CAS Key Laboratory of Innate Immunity and Chronic Disease, Division of Life Sciences and Medicine, University of Science and Technology of China, Hefei, Anhui 230027 China; 7 Institute of Biopharmaceutical and Health Engineering, Tsinghua Shenzhen International Graduate School, Tsinghua University, Shenzhen, 518055 PR China

**Keywords:** Endometrial cancer, Targeted therapies

## Abstract

Loss of phosphatase and tensin homolog (PTEN) impairs DNA double-strand repair and confers sensitivity to poly (ADP-ribose) polymerase inhibitors (PARPis). However, PARPis also hyperactivate the MAPK and PI3K/AKT/mTOR pathways in PTEN-*deficient* endometrial carcinoma (EC), which allows the emergence of PARPi resistance. BCL-2–associated death promoter (BAD), integrates the common cell survival effects of the RAS/MEK/MAPK and PI3K/AKT/mTOR pathways. Herein, it was observed that increased BADSer99 (BADS99) phosphorylation in EC cells was significantly associated with *PTEN*-*deficient* status. Forced expression of phosphorylation deficient human BADS99A in PTEN-*deficient* EC cells significantly increased CASPASE 3/7 activity and decreased EC cell viability. Using NPB as a pharmacological inhibitor of pBADS99 phosphorylation, it was demonstrated that NPB synergized with PARPis (Olaparib, Rucaparib and Talazoparib) to enhance PARPi IC_50_ up to 60-fold and decreased survival, foci formation, and growth in 3D ex vivo culture of *PTEN*-*deficient* EC cells. Combined NPB-PARPi treatment of PTEN-*deficient* EC cells stimulated apoptosis and promoted DNA damage by impairment of homologous recombination. Using the *clustered regularly interspaced short palindromic repeats (CRISPR)-Cas9 endonuclease* system it was demonstrated that deletion of *PTEN* in PTEN replete EC cells enhanced the efficacy of combined NPB-PARPi treatment. Furthermore, combined inhibition of BADS99 phosphorylation and PARP ablated xenograft growth of PTEN-*deficient* EC cells. Similarly, a combination of NPB and PARPis significantly suppressed the growth of PTEN deficient patient-derived EC organoids. Hence, combined inhibition of BADS99 phosphorylation and PARP represents a rational and efficacious strategy to improve the prognosis of recurrent EC patients.

## Introduction

Loss of Phosphatase and tensin homolog (*PTEN*) function is positively associated with endometrial carcinoma (EC) progression [1]. *PTEN* is a tumor suppressor gene encoding a phosphatase, which negatively regulates the PI3K/AKT/mTOR (PAT) pathway, contributing to EC cell survival, progression, and resistance to therapy [2, 3]. PTEN also contributes to the maintenance of genomic integrity in cells through upregulation of RAD51 expression [4]. *PTEN*-deficient EC cells consequently exhibit loss of RAD51-associated functions and deficient homologous recombination (HR) resulting in impaired DNA double-strand break (DSBs) repair [5].

The synthetic lethal interaction between poly (ADP-ribose) polymerase (PARP) inhibition and loss of breast cancer (*BRCA*) gene 1/2 functions has been clinically applied. PARP inhibitors (PARPis) were developed to treat breast, ovarian, prostate, and pancreatic cancers with *BRCA1/2* mutation in which HR is impaired [6]. However, not only *BRCA1/2* mutated cancers are sensitive to PARPis; cancers carrying mutations impairing DNA HR, such as *PTEN* mutations, are also sensitive to PARP inhibition [7]. Indeed, investigations have also demonstrated increased sensitivity of *PTEN*-deficient EC cells to PARPis [8–10]; and PARPis, such as Olaparib or Talazoparib have shown efficacy in *PTEN*-deficient EC [11, 12]. Moreover, both *PTEN*-deficiency or PARP inhibition in cancer cells activates the PAT pathway [13, 14] and inhibition of the PAT pathway has been demonstrated to sensitize cancer cells to PARPis [15, 16]. Combined Olaparib and BKM120 (a PI3K inhibitor) treatment of *PTEN*-deficient EC has been reported to improve efficacy compared to single-agent treatment [17]. Phase-I trials combining Olaparib with AZD2014 (a mTORC1/2 inhibitor) or AZD5363 (an AKT inhibitor) in advanced EC (NCT02208375) also reported a superior combination treatment efficacy [18, 19]. However, PARPi associated off-target activity [20–23], hyperactivation of PAT signaling, and trans-hyperactivation of RAS/MEK/MAPK (RMM) pathway through feedback loops of PI3K-MAPK signaling [24, 25] produce resistance to therapy [26–28] in EC patients (tabulated in the supplementary information, SI 1) and significantly hinder the clinical utility of PARPis [25, 29, 30]. Thus, the identification and development of novel effective therapeutic strategies targeting pivotal effectors of the PAT pathway may improve the prognosis of EC.

The BCL-2–associated death promoter (BAD) protein is a BH3-only member of the BCL-2 family of proteins that integrates the common cell survival effects of the PAT and RMM pathways [31, 32]. Human (h) BAD is phosphorylated (p) at Serine (S)75 primarily through p44/42 MAP kinase pathway activation [33], and at S99 primarily through the activation of AKT/p70S6K [34, 35], although other kinases may also phosphorylate these residues. pBAD is bound to 14-3-3 protein and is thus unavailable to sequester BCL-2, BCL-XL, or BCL-W to subsequently promote apoptosis [36]. Hence, inhibition of both the PAT and RMM pathways leads to reduced pBAD levels and ultimately activates the pro-apoptotic functions of BAD [37]. Elevated BAD phosphorylation is associated with the progression of various human malignancies including EC [38]; and increased pBAD (S75/99) phosphorylation is positively associated with decreased apoptosis in EC compared to normal endometrial specimens [39]. The efficacy of a novel small molecule inhibitor of BADS99 phosphorylation, NPB [40], in combination with cisplatin, has been recently reported in ovarian carcinoma [41]. Herein, the potential therapeutic synergy between NPB and PARPis in *PTEN*-deficient EC has been determined in preclinical models.

## Materials and methods

### Cell culture and reagents

KLE (*PTEN*-proficient) and Ishikawa, RL-95-2, or AN3CA (*PTEN*-deficient) EC cell lines were purchased from Procell Life Science & Technology Co. Ltd (Wuhan, China). Clinicopathological features, molecular profiles, short tandem repeat information, and culture conditions of EC cell lines are tabulated in SI 2A. Details of the *sgPTEN*-*pSpCas9(BB)-2A-Puro (PX459) V2.0* construct are summarized in SI 2B. *hBAD or hBADS99A* (Ser99 mutated to Ala99) construct with a flag-tag is described in SI 2F. Based on the PARP-trapping capacities [42–45] (SI4A), three PARPis, Olaparib (specific to PARP1/2). Rucaparib (pan-PARP) or Talazoparib (specific to PARP1) were utilized. Olaparib (AZD2281), Rucaparib (AG-014699), and Talazoparib (BMN 673) were purchased from SelleckChem (Houston, TX, USA). All experiments were performed in 2% FBS with the respective medium.

### Western blot (WB) and HR reporter assay analysis

WB analysis was performed as previously described [40, 46, 47] using the primary and secondary antibodies described in SI 2C. HR reporter assay and sensitivity assays and the stable clones of AN3CA-*hprtDR-GFP* were generated as previously described [48]. Briefly, after 48 h of pre-treatment with NPB or vehicle, AN3CA-*DR-GFP* (1 × 10^6^) cells were transfected by FuGENE6 (Promega, US) with 2 μg *pCMV-I-SceI*, respectively. The cells were assessed for green fluorescence emission using flow cytometry.

### Oncogenic and immunofluorescence (IF) analyses

AlamarBlue® viability, total cell count, foci formation, suspension culture, and growth in 3D Matrigel culture were performed as previously described [40, 47]. Biochemical assays, cell viability, apoptosis, and cytotoxicity were performed using ApoTox-Glo™ Triplex Assay Kit (G6320, Promega, China) as previously described [40]. Phosphatidylserine exposure and cell death, Live/Dead cell visualization, and IF analysis was performed as described previously [40, 49]. Information of primary/secondary antibodies utilized are tabulated in SI 2C. Nuclei were stained in a mounting medium with DAPI (ab104193, Abcam). Combination index (CI) analysis was performed using the Chou-Talalay CI method [50].

### In vivo and patient-derived EC organoid (PDECO) analysis

*Xenograft* studies were performed as previously described [40, 46, 47] (SI 2D); and patient-derived EC cells and organoid culture and treatment were performed as described in SI 2E. All assays of this study were approved by the Laboratory Animal Ethics Committee (Certificate number: YW). Fresh human EC tissues were obtained with written informed consent and approval from the patients and the Ethical Committee of the University of Hong Kong-Shenzhen Hospital (HKU-SZH, research No. hkuszh2019105, approval No. [2019]096, Date: 2019.03.26. Detailed information is summarized in SI 2E.

### Statistical analysis

Statistical analysis was performed using SPSS 25 (IBMSPSS Statistics, IBM Corp., Armonk, NY, USA) and GraphPad Prism 7.0 (GraphPad Software, San Diego, CA, USA) as previously described [40]. For in vitro assays, the statistical differences among subgroup analyses were compared using an unpaired two-tailed Student *t* test. For in vivo assays, the statistical differences between the treatment groups were compared using a one-way ANOVA followed by a Tukey’s multiple comparison test. *p-*values < 0.05(*), *p* < 0.01(**) and *p* < 0.001(***) were considered statistically significant. Quantitative data are expressed as mean ± SD, unless otherwise stated.

## Results

### Inhibition of BADSer99 phosphorylation decreases cell survival of *PTEN*-deficient EC cells

To examine the functional relevance of pBADS99/BAD in the regulation of EC survival, the effect of BADSer99 mutation on cell survival was examined. Forced expression of *Flag-hBADS99A* into AN3CA cells significantly decreased the levels of pBADS99/BAD compared to control cells (SI 3A). A marginal increase in the levels of pBADS75/BAD was observed in AN3CA cells with forced expression of *Flag-hBADS99A* compared to control cells. Forced expression of *Flag-hBADS99A* in AN3CA cells resulted in significantly increased levels of total BAD protein compared to control cells. Functionally, forced expression of *hBADS99A* in AN3CA cells significantly increased CASP3/7 activity and decreased cell viability compared to control AN3CA cells (SI 2A). Thus, pBADS99 is required for the survival of *PTEN*-deficient EC cells.

### NPB synergizes with PARPis in EC cells to decrease survival

**Fig. 1:**
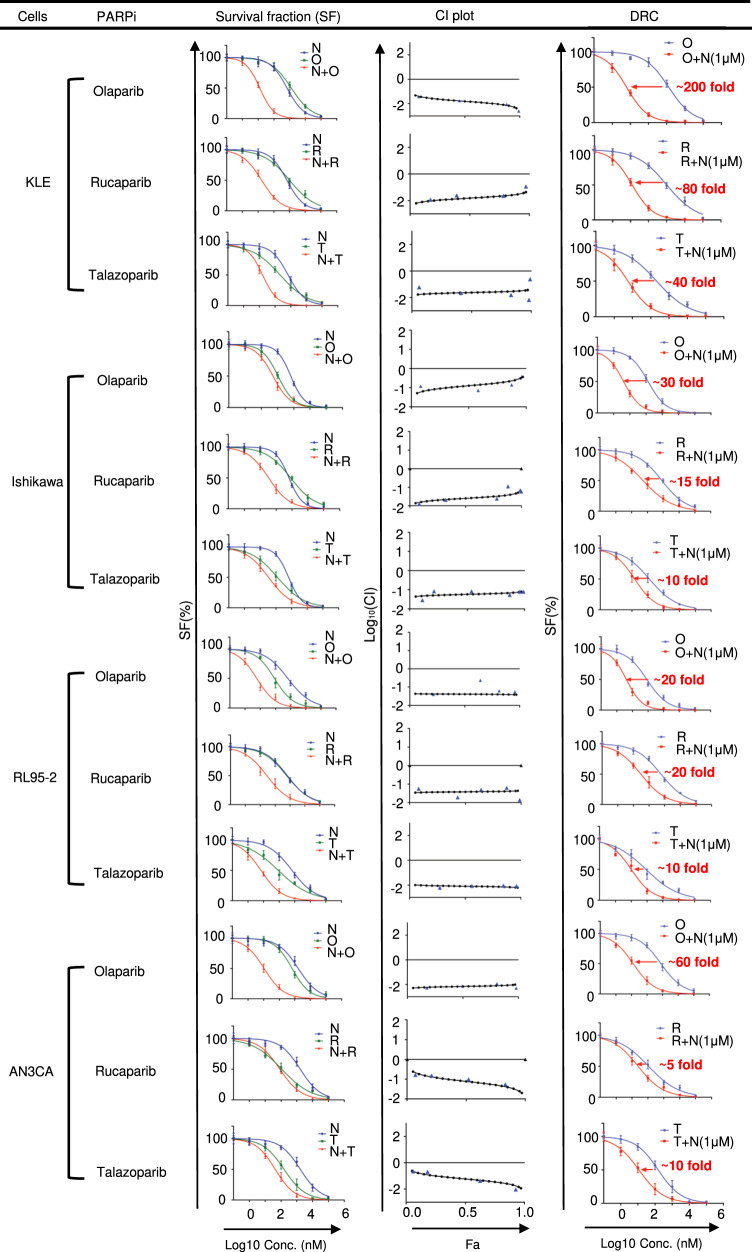
NPB synergizes with PARPis to decrease EC cell survival. Chou-Talalay synergy analysis for NPB (N) in combination with three PARP inhibitors, namely Olaparib (O), Rucaparib (R) or Talazoparib (T), in a panel of EC cells including KLE (*PTEN-*proficient), AN3CA (*PTEN-*deficient), Ishikawa (*PTEN-*deficient) and RL95-2 (*PTEN-*deficient). Cells were treated with the indicated concentration (log_10_ scale) of NPB and mentioned PARP inhibitor for 6 days. The survival fraction was assessed using a total cell number assay. The logarithmic combination index (CI) value corresponding to cell fraction affected (Fa) was determined using the CompuSyn software (http://www.combosyn.com) as described in “Materials and methods”. CI value indicates: <1 synergism; =1 additive Synergy; >1 antagonism (*n* = 3). Dose-response curves for a panel of cells treated with the indicated concentration of PARP inhibitors with or without 1 µM NPB in total cell number assays. Arrow indicates fold reduction in respective PARP inhibitor IC_50_ in the presence of NPB (*n* = 3).

The potential synergy between pharmacological inhibition of BADS99 phosphorylation and PARPis in EC was next assessed. *PTEN*-proficient KLE cells exhibited higher IC_50_ values for the three PARPis compared to *PTEN*-deficient EC cells (Fig. 1 and SI.4B). Based on combination index (CI) analysis, NPB exhibited a strong synergistic combination with PARPis in all four EC cell lines. Combined NPB (1µM)-PARPi treatment of EC cells significantly increased the efficacy of PARPis compared to single PARPi treatment, as demonstrated using dose-response analysis. Notably, combined treatment of KLE cells with NPB-Olaparib resulted in a ~200-fold decrease in Olaparib IC_50_ compared to Olaparib alone. Moreover, combined treatment of Ishikawa cells with NPB-Olaparib demonstrated a decreased IC_50_ by ~30-fold, in RL95-2 by ~20-fold, and in AN3CA by ~60-fold compared to Olaparib alone (Fig. 1). Similar directional fold-changes were also observed with combined treatment of NPB with Rucaparib or Talazoparib in all four EC cell lines. Thus, NPB synergized with PARPis in EC cells to decrease cell survival.

### NPB synergizes with PARPis to inhibit foci formation and 3D Matrigel growth of EC cells

**Fig. 2:**
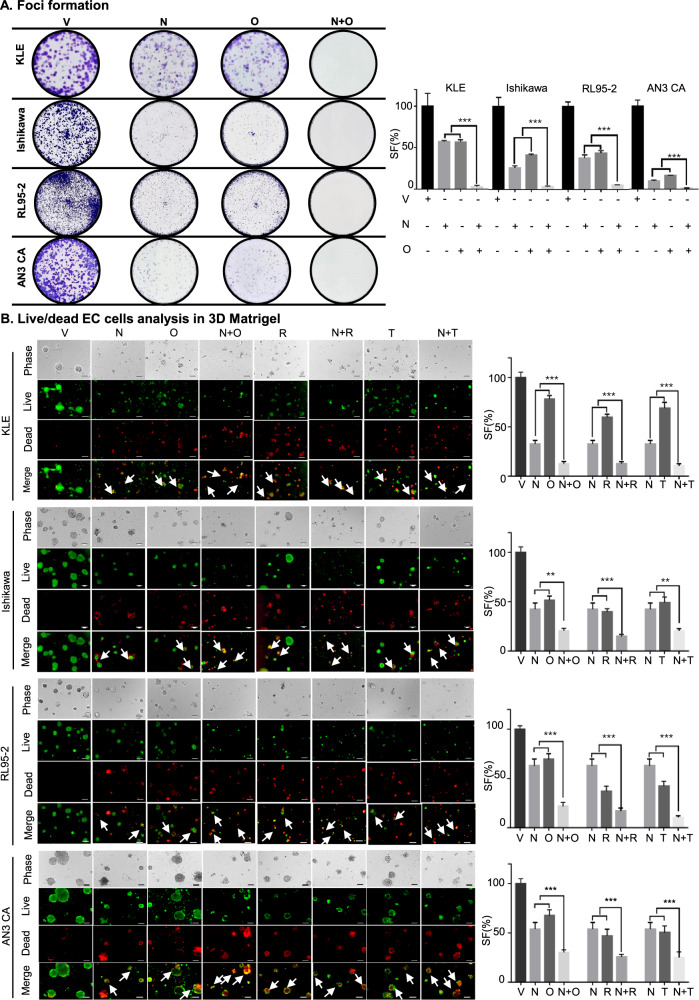
NPB synergizes with PARPis to inhibit foci formation and 3D growth of EC cells. **A** EC cells, KLE, AN3CA, Ishikawa, and RL95-2 were incubated with the indicated drug concentrations of NPB (N) and Olaparib (O), Rucaparib (R) or Talazoparib (T) or combinations thereof for foci formation assays and stained with 0.2% crystal violet (*n* = 3). **B** Microscopic visualization of calcein-AM (green) stained spheroids (live) and BOBO-3 Iodide (red) stained cell debris (dead) generated by EC cells (KLE, Ishikawa, RL95-2, and AN3CA) cultured in 3D Matrigel after exposure to NPB (N) and PARP inhibitors (Olaparib = O, Rucaparib = R, Talazoparib = T) and combinations thereof (*n* = 3). Red-fluorescence (staining of ethidium homodimer-1 indicating loss of plasma membrane integrity) and decreased green-fluorescence (staining of calcein-AM to indicate intercellular esterase activity). Scale bars, 100 µm. Right: Cell fraction (SF) was evaluated using AlamarBlue® in EC cells treated with NPB (N), PARP inhibitors (Olaparib = O, Rucaparib = R, Talazoparib = T) and in combination with the indicated drug concentrations for 14 days in 3D Matrigel. (*n* = 3). The white color arrow highlights the apoptotic cell death. Columns are mean of triplicate experiments; bars, ±SD. *p < 0.05, **p < 0.01, ***p < 0.001.

The treatment of AN3CA cells with either single-agent NPB/Olaparib or combined NPB-Olaparib significantly attenuated the capacity for foci formation as compared to vehicle-treated AN3CA cells (Fig. 2A). Combined NPB-Olaparib treatment of AN3CA cells completely abolished the capacity for foci formation compared to NPB or Olaparib alone. In 3D Matrigel culture, the treatment of pre-grown AN3CA colonies with NPB or Olaparib singly, or combined NPB-Olaparib produced markedly increased red-fluorescence (indicating apoptotic cells) and decreased green-fluorescence (indicating live cells) compared to vehicle-treated AN3CA colonies (Fig. 2B). Combined NPB-Olaparib treatment of AN3CA colonies significantly augmented red-fluorescent staining and reduced green-fluorescent staining compared to either NPB or Olaparib treated AN3CA colonies. Also, treatment of AN3CA colonies with combined NPB-Olaparib produced significantly decreased cell viability compared to NPB or Olaparib treated AN3CA colonies in Matrigel (Fig. 2B, right side). Similar directional changes in foci formation and apoptotic cell death in 3D Matrigel was observed in KLE, Ishikawa, and RL95-2 cells after combined treatment of NPB with PARPis (Fig. 2).

### NPB synergizes with PARPis in EC cells to stimulate intrinsic apoptosis through promoting DNA damage and impairing DNA repair

**Fig. 3:**
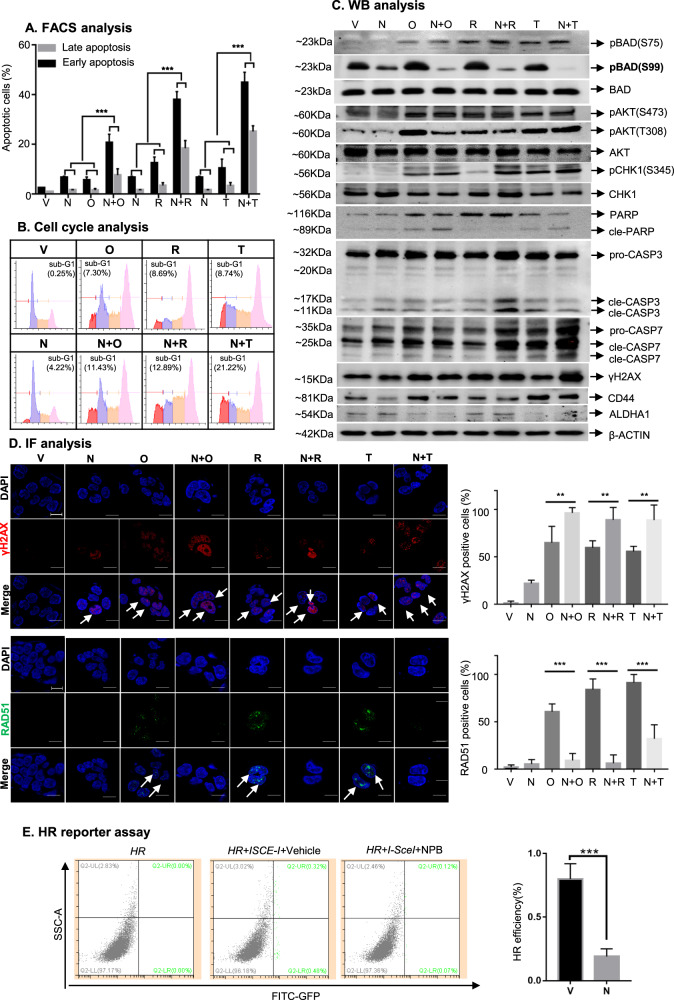
NPB synergizes with PARPis in AN3CA cells to stimulate apoptosis by promoting DNA damage and impairing DNA repair. **A** Flow cytometric analysis of Annexin-V and propidium iodide (PI) staining of apoptotic cell death in AN3CA cells measured after treatment with NPB (N) and Olaparib (O), Rucaparib (R) or Talazoparib (T) or combinations at 72 h as described in materials and methods. Apoptotic cells refer to annexin-V positive (early apoptosis) + annexin-V and PI double-positive (late apoptosis) cells (*n* = 3). **B** Representative flow cytometry plots using PI staining of DNA for AN3CA cells measured after treatment with NPB (N) and Olaparib (O), Rucaparib (R) or Talazoparib (T) or combinations thereof at 72 h as described in “Materials and methods” (*n* = 3). **C** Western blot analysis was used to assess the expression of various proteins and the level of protein phosphorylation or cleavage in AN3CA cells after treatment with NPB (N), Olaparib (O), Rucaparib (R), and Talazoparib (T) or combinations thereof. Soluble whole-cell extracts were run on an SDS-PAGE and immunoblotted as described in materials and methods. β-ACTIN (ACTB) was used as input control for cell lysate. The sizes of detected protein bands in kDa are shown on the left side. **D** Representative immunofluorescent images of DNA damage markers γH2AX and RAD51 in AN3CA cells. Cells were treated with NPB (N) Olaparib (O), or a combination (N + O). Scale bars, 50 µm. Right: Semi-quantification of γH2AX and RAD51 foci in AN3CA cells treated with NPB (N), Olaparib (O), or a combination (N + O) are shown. Cells with more than 5 RAD51 foci per nucleus were counted as RAD51 positive cells. 100 cells were analyzed in 2–3 separate fields for each sample (*n* = 3). Representative EC cells with positive γH2AX staining and RAD51 foci are highlighted using white color arrows. **E** Flow cytometric analysis of pHPRT-DRGFP transfected AN3CA cells (HR). Cells were transfected with I-SceI expressing vector to induce DSBs which may be repaired by HR using iGFP as a template and restore a functional *GFP* gene. The percentage of GFP-positive (GFP+) cells was measured using immunofluorescence and flow cytometry as an indicator of HR efficiency. Right: Quantification of HR efficiency as assessed by GFP signal in AN3CA cells. *p < 0.05, **p < 0.01, ***p < 0.001. Columns are the mean of samples in each group; bars, ±SD. *p < 0.05, **p < 0.01, ***p < 0.001.

The mechanistic basis for the synergistic effects of combined NPB-PARPi treatment was next elucidated in AN3CA cells (SI2A). The treatment of AN3CA cells with either a single agent or combined NPB-Olaparib resulted in an increased population of early and late apoptotic cells compared to vehicle-treated AN3CA cells (Fig. 3A, SI5A). Combined NPB-Olaparib treatment of AN3CA cells produced a significantly augmented population of early and late apoptotic cells compared to AN3CA cells treated with either NPB or Olaparib alone (Fig. 3A, SI5A). The combined NPB-Olaparib treatment of AN3CA cells resulted in increased cell populations in the sub-G1-phase compared to AN3CA cells treated with either vehicle or NPB alone (Fig. 3B, SI5B). Similar directional changes in apoptosis and sub-G1-phase cell population of AN3CA cells were also observed after the combined treatment of NPB with Rucaparib or Talazoparib.

NPB treated AN3CA cells exhibited decreased levels of pBADS99, whereas, no changes were observed in pBADS75, pAKT (T308 & S473) or AKT protein levels compared to vehicle-treated AN3CA cells as demonstrated using WB analysis (Fig. 3C, SI6). In contrast, Olaparib treatment of AN3CA cells exhibited an increased pAKT-T308, pAKT-S473, and pBADS99 levels compared to vehicle-treated AN3CA cells. Concomitantly, Olaparib treatment of AN3CA cells also exhibited increased pBADS75 levels, indicative of the activation of RMM signaling [33]. Combined NPB-Olaparib treated AN3CA cells exhibited significantly decreased pBADS99 levels compared to AN3CA cells treated with either NPB or Olaparib alone (Fig. 3C, SI 6). No changes were observed in AKT or BAD protein levels in AN3CA cells after treatment with either a single drug or combined NPB-Olaparib. Similar directional changes in pAKT/AKT and pBAD/BAD levels were observed in AN3CA cells after treatment with NPB alone or combined with Rucaparib or Talazoparib.

PARPis-induced DNA damage (SSB) [51] activates CHK1 phosphorylation, thereby arresting cell cycle progression and allowing DNA damage repair and replication [52]. pCHK1 (at S345) [53] exhibited no apparent change in AN3CA cells with NPB treatment compared to vehicle-treated AN3CA cells (Fig. 3C, SI6). However, AN3CA cells treated with Olaparib significantly increased pCHK1-S345 levels compared to NPB or vehicle. Combined NPB-Olaparib treatment of AN3CA cells exhibited marginally increased pCHK1-S345 levels compared to AN3CA cells treated with Olaparib alone (Fig. 3C, SI6). Olaparib treatment of EC cells also has been reported to increase γH2AX, and RAD51 expression, an indicator of HR repair in EC cells [54]. Treatment of AN3CA cells with either NPB or Olaparib resulted in elevated γH2AX protein levels compared to vehicle-treated AN3CA cells, as demonstrated using IF analysis. Notably, AN3CA cells treated with Olaparib exhibited higher γH2AX levels compared to NPB treatment (Fig. 3D). Compared to Olaparib treatment alone, combined NPB-Olaparib treatment of AN3CA cells resulted in elevated γH2AX foci in nuclei and reduced nuclear RAD51 foci formation (Fig. 3D). Similar directional changes in pCHK1-S345/CHK1 protein levels and γH2AX/RAD51 foci formation were observed in AN3CA cells after NPB treatment with either Rucaparib or Talazoparib (Fig. 3C, D, SI 6). In addition, NPB treatment of AN3CA cells also suppressed HR efficiency as demonstrated using HR reporter assays (Fig. 3E, SI8).

Furthermore, the treatment of AN3CA cells with either NPB or Olaparib alone resulted in increased cleaved-CASP3/7, cleaved-PARP, and γ-H2AX protein levels compared to vehicle-treated AN3CA cells (Fig. 3C). Combined NPB-Olaparib treatment of AN3CA cells exhibited marginally increased cleaved-CASP3/7, cleaved-PARP, and γ-H2AX levels compared to either vehicle or single NPB/Olaparib treated AN3CA cells. Notably, no increase in the cleaved-PARP subunit was detected after treatment with Rucaparib; whereas, combined NPB-Rucaparib treatment significantly increased cleaved-CASP3/7 levels compared to either vehicle or single NPB or Rucaparib treated AN3CA cells. Compared to AN3CA cells treated with either NPB or PARPis alone, increased cleaved-CASP3/7 levels were observed in NPB-Rucaparib and increased cleaved-CASP7 in NPB-Talazoparib treated cells, respectively. These differences are presumably to the diverse pharmacological properties [55] and PARP-trapping capacities described in SI4B. Akin to a previous observation [41], decreased CD44 and ALDHA1 protein levels were also observed in AN3CA cells after NPB treatment compared to vehicle-treated cells. However, an increase in CD44 and a decrease in ALDHA1 levels were observed in AN3CA cells after Olaparib treatment compared to vehicle. Combined NPB-Olaparib treatment of AN3CA cells attenuated the increased CD44 levels compared to Olaparib-treated cells (Fig. 3C, SI7). Similar directional changes were observed in cleaved-CASP3/7, PARP, CD44, and ALDHA1 protein levels in AN3CA cells after NPB treatment combined with either Rucaparib or Talazoparib. Therefore, NPB synergizes with PARPis in AN3CA cells by promoting DNA damage via impairment of DNA repair with subsequent stimulation of apoptosis.

### *PTEN* deletion in KLE cells sensitizes to combined NPB-PARPi treatment efficacy

**Fig. 4:**
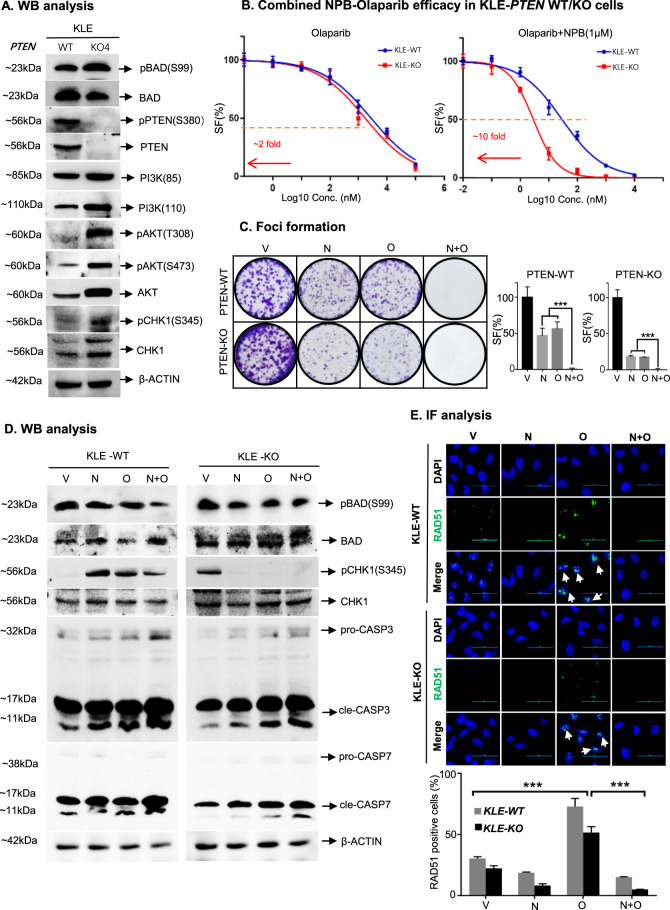
*PTEN-*deletion sensitizes KLE cells to combined NPB-PARPi treatment. **A** Western blot analysis was used to assess the expression of PTEN and downstream effectors PI3K, AKT, BAD, and CHK1 protein; and levels of pAKT, pBADS99, and pCHK1 were determined in KLE- WT, and PTEN-KO cells. Soluble whole-cell extracts were run on an SDS-PAGE and immunoblotted as described in materials and methods. β-ACTIN was used as input control for cell lysates. The sizes of detected protein bands in kDa are shown on the left side Densitometries of protein bands were subsequently determined using ImageJ software (https://imagej.nih.gov/ij/). **B** Dose-response curves for a panel of KLE-WT and KLE-PTEN-KO cells treated with the indicated concentration of Olaparib with or without a constant concentration of NPB (1μM) in total cell number assays. Arrow indicates fold reduction in respective PARP inhibitor IC_50_ in the presence of NPB (*n* = 3). **C** KLE-WT and KLE-PTEN-KO cells were incubated with the indicated concentrations of NPB (N), Olaparib (O), or a combination thereof for foci formation and stained with 0.2% crystal violet (*n* = 3). **D** Western blot analysis was used to assess the expression of BAD, CHK1, cle-CASP, and cle-CASP7 protein and the level of pBADS99 in KLE-WT and KLE-PTEN-KO cells after treatment with NPB (N), Olaparib (O) or combination thereof. Soluble whole-cell extracts were run on an SDS-PAGE and immunoblotted as described in materials and methods. β-ACTIN (ACTB) was used as input control for cell lysate. The sizes of detected protein bands in kDa are shown on the left side. **E** Representative immunofluorescent images of RAD51 in KLE-WT and KLE-PTEN-KO cells. Cells were treated with NPB (N), Olaparib (O), or a combination (N + O). Scale bars, 50 µm. Below: Quantification of RAD51 foci positive AN3CA cells treated with NPB (N), Olaparib (O), or a combination (N + O) are shown. Cells with more than 5 RAD51 foci per nucleus were counted as RAD51 positive cells. 100 cells were analyzed in 2–3 separate fields for each sample (*n* = 3). Representative EC cells with positive RAD51 foci are highlighted using the white color arrow. Columns and points are the mean of triplicate determinations; bars, ±SD. *p < 0.05, **p < 0.01, ***p < 0.001.

Next, the functional relevance of PTEN function on the combined NPB-PARPi treatment of EC cells was elucidated. A KLE *PTEN*-knockout (KLE-KO) cell line was established as described in materials and methods (SI9A). KLE-KO cells exhibited elevated pAKT-S473, pAKT-T308, and pBADS99 levels compared to KLE-WT cells (Fig. 4A, SI9B), indicating hyperactivity of the PAT pathway in KLE-KO cells after *PTEN-*deletion. Similar directional differences in the PAT pathway were also observed between *PTEN-deficient* and *PTEN*-*WT* EC cell lines (SI 3B).

The IC_50_ value of Olaparib reduced 1~3-fold and the IC_50_ value of combined Olaparib-NPB reduced 10~15-fold in KLE-KO cells compared to KLE-WT cells (Fig. 4B, SI 9C). Also, the capacity of KLE-KO cells to form foci on monolayer culture was significantly reduced compared to KLE-WT after treatment with similar doses of Olaparib, NPB, or combined NPB-Olaparib (Fig. 4C). Similar to the effects observed in AN3CA cells, data here indicated a synergistic effect of NPB and Olaparib. *PTEN*-deletion in KLE cells sensitized the efficacy of not only NPB or Olaparib alone but also combined NPB-Olaparib treatment. Similar directional changes in the functional efficacy were observed in KLE-KO cells treated with Rucaparib or Talazoparib (Figs. 4B, C, SI 9C).

The mechanism of the sensitization induced by *PTEN deletion* was next assessed using WB analysis. Both in KLE-WT and KLE-KO cells, treatment with NPB alone decreased the pBADS99 levels (Fig. 4D). Also, combined NPB-Olaparib treatment of both KLE-WT and KLE-KO cells further decreased the pBADS99 levels; whereas no change was observed in BAD protein levels after treatment (Fig. 4D). Treatment of KLE-WT cells with either NPB or Olaparib resulted in increased pCHK1-S435 levels compared to vehicle-treated cells; whereas, combined NPB-Olaparib treatment of KLE-WT cells exhibited decreased pCHK1-S435 levels compared to KLE-WT cells treated with NPB or Olaparib alone (Fig. 4D). In contrast, pCHK1-S435 was not detected in KLE-KO cells after treatment with either NPB/Olaparib or combined NPB-Olaparib (Fig. 4D). Only vehicle-treated KLE-KO cells exhibited phosphorylation of CHK1-S435. No significant change was observed in CHK1 protein levels in either KLE-WT or KLE-KO cells. As observed previously (Fig. 3C), both KLE-WT and KLE-KO cells treated with either NPB or Olaparib exhibited increased cleaved-CASP3/7 levels compared to vehicle-treated cells (Fig. 4D). Moreover, combined NPB-Olaparib treatment of either KLE-WT or KLE-KO cells further increased the cleaved-CASP3/7 levels compared to cells treated with NPB or Olaparib alone (Fig. 4D). Of note, *PTEN-deletion* results in enhanced activation of the PAT pathway and increased levels of pBADS99 [34, 35, 56]. Herein, elevated pBADS99 levels were observed in KLE-KO compared to KLE-WT cells (Fig. 4A, D, SI 9B and 10). The treatment of KLE-KO cells with NPB produced significantly decreased pBADS99 and increased cleaved-CASP3/7 levels compared to KLE-WT cells treated with NPB alone (Fig. 4D, SI10), indicating that *PTEN-*deletion enhances NPB efficacy in KLE-KO cells. As PTEN functions in HR [4, 10], RAD51 foci formation in the nuclei of KLE-WT/KO cells was examined after either NPB, Olaparib, or NPB-Olaparib combination treatment. Compared to KLE-WT, KLE-KO cells exhibited significantly decreased RAD51 foci after Olaparib treatment. Also, consistent with that observed in AN3CA cells, Olaparib-induced RAD51 foci were abolished in KLE-KO cells after combined NPB-Olaparib treatment (Fig. 4E).

### Combined NPB-PARPi treatment suppresses the growth of EC xenografts

**Fig. 5:**
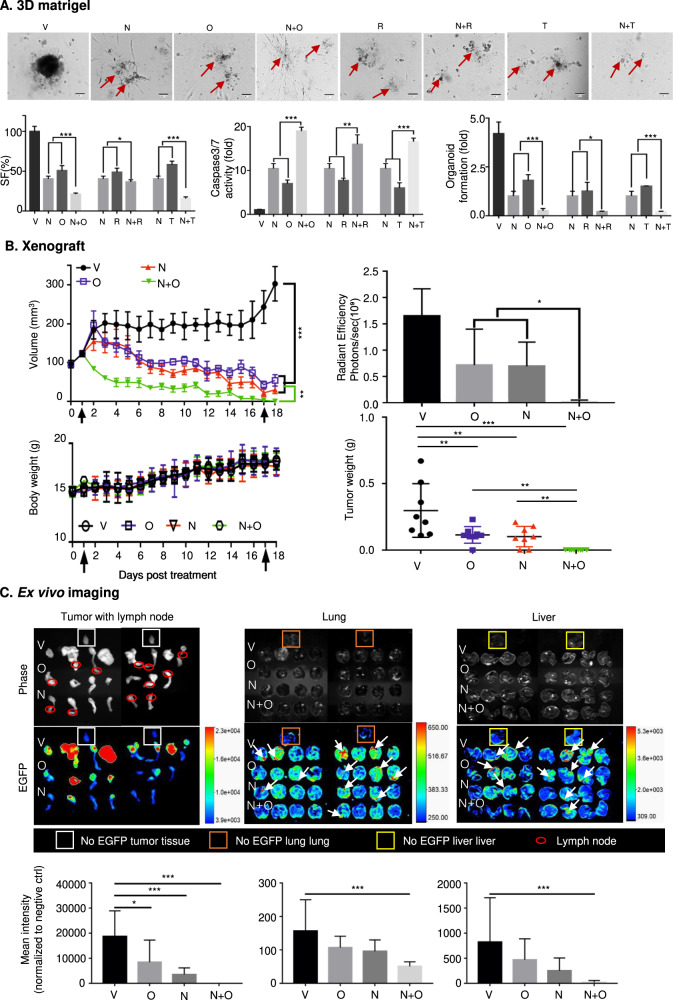
Combined NPB-PARPi treatment suppresses the growth of AN3CA-GFP-derived xenografts and patient-derived EC organoids (PDECO) in ex vivo culture. **A** AN3CA-EGFP (5 × 10^6^) cells were injected subcutaneously into the flank of 5-week-old BALB/c athymic mice. When the xenograft reached ~100 m^3^ the mice were randomized into the 4 indicated treatment groups (*n* = 8 each group). Mice were treated daily with vehicle (V), 20 mg/kg NPB (N), 50 mg/kg Olaparib (O), or a combination of NPB-Olaparib by i.p. injection. Left upper: xenograft growth was monitored daily by measurement of volume. Left lower: Mean weights of animals in each treatment group are indicated. Animal weight was monitored daily. Arrows indicate the start and the end of the treatment. Right upper: bioluminescence subcutaneous surface of AN3CA-EGFP xenograft in each group was observed using a fluorescence imaging system (IVIS Spectrum, PerkinElmer, US) on the 18^th^ day after the start of injection. Right lower: Mean xenograft weight of each treatment group after sacrifice. Results represent the mean ± SEM of eight animals. **B** Representative images of bioluminescence detection for lymph node, lung, and liver regions. EGFP negative animal organ tissue was used as a negative control. Bioluminescence intensity was quantified for the evaluation of metastases. **C** Effects of NPB (N), PARP inhibitors (Olaparib = O, Rucaparib = R, Talazoparib=T), or combination treatment for 9 days in EC patient-derived organoids (PDOs) was evaluated using the ApoTox-Glo Triplex Assay Kit for survival fraction (SF) and CASPASE3/7 activity. Organoid growth was determined by the counting of the number of organoids with diameters >100 µm number on the 7th day of culture; Scale bars: 100 µm. Days in red color meant drug administration on that day. Red color arrow highlights the apoptotic PDOs. Columns are mean of samples in each group; bars, ±SD. *p < 0.05, **p < 0.01, ***p < 0.001.

Xenografts were generated by subcutaneous injection of AN3CA cells with stable expression of EGFP (AN3CA-GFP) into immunocompromised mice as described in SI11A&B. Xenograft-bearing (~100 mm^3^) mice were randomly grouped (*n* = 8) and were injected i.p. with the vehicle, Olaparib (50 mg/kg), NPB (20 mg/kg), or the combination of NPB and Olaparib daily at the same respective concentrations. The treatment regimen is summarized in SI11A. AN3CA-GFP cell-generated xenograft volumes and animal body weights were measured daily as represented in Fig. 5B. Xenografts derived from AN3CA-EGFP cells exhibited a significant reduction in xenograft volumes in all treated groups (Olaparib, NPB, or Olaparib-NPB) after 3-days compared to vehicle-treated animals. The combined NPB-Olaparib treated group of animals exhibited significantly further decreased xenograft volume compared to treatment with either NPB or Olaparib alone. Seventeen days after commencement of drug treatment, complete ablation of xenografts was observed in animals treated with the NPB-Olaparib combination. On the 18th day, animals from all groups were sacrificed. No significant changes in animal body weight were observed in treatment groups compared to a vehicle-treated group during the treatment period. All treated groups from AN3CA-GFP cell-derived xenografts (NPB, Olaparib, and NPB-Olaparib) exhibited decreased xenograft weight as compared to the vehicle-treated group. The weight of ablated xenografts was considered zero for the statistical analysis. The NPB treated group of animals exhibited reduced xenograft weight compared to the Olaparib treated animal group, although the change was not statistically significant. In vivo fluorescence imaging of live animals positively correlated with the AN3CA-GFP cell-derived generated xenograft volume and xenograft weight (SI 11C). The combined NPB-Olaparib treated animal group exhibited no notable GFP signal compared to either vehicle or single agent (NPB or Olaparib) treated animal groups. Detectable GFP signals were observed in the lung, liver, and lymph nodes of different animals indicating metastases to these organs (Fig. 5C). Representative fluorescence and bright-field images of metastatic nodules in lymph nodes, lungs, and liver are represented in Fig. 5C. 2/8 animals in the vehicle-treated group and 2/8 animals in the Olaparib-treated group demonstrated metastatic nodules in lymph nodes. Notably, no metastatic nodules in lymph nodes were observed in the group of animals treated with either NPB or combined NPB-Olaparib. 4/8 animals in the vehicle-treated group, 4/8 animals in Olaparib treated group, 3/8 animals in the NPB treated group, and 1/8 animals in the combined NPB-Olaparib treated group demonstrated metastatic nodules in the lung. 4/8 animals in the vehicle-treated group, 4/8 animals in the Olaparib treated group, 2/8 animals in the NPB treated group, and 1/8 animals in the combined NPB-Olaparib treated group demonstrated metastatic nodules in the liver (Fig. 5C). Thus, combined NPB-Olaparib treatment ablated xenograft growth and significantly reduced the metastatic capacity of EC xenografts.

### Combined NPB-PARPi treatment suppresses the growth of PDECOs in ex vivo culture

We next established PDECOs from the resected specimens of primary *PTEN*-deficient endometrioid EC as described in SI11 [57]. The effect of combined NPB-PARPi treatment in PDECOs was determined. Both NPB and Olaparib alone decreased PDECO cell survival. Compared to either Olaparib or NPB alone, combined NPB-Olaparib treatment of PDECOs produced significantly further decreased cell survival. In addition, combined NPB-Olaparib treatment of PDECOs exhibited significantly increased CASP3/7 activities and decreased growth of organoids compared to PDECOs treated with NPB or Olaparib alone. Similar directional changes in cell survival, CASP3/7 activities, and capacity for PDECO growth were also observed with combined treatment of NPB with either Rucaparib or Talazoparib.

## Discussion

Despite recent advancements in EC treatment, the prognosis for high-grade EC is poor with a 5-year OS rate of <17%. Therapy resistance and recurrence of disease largely contribute to the mortality rate of patients [58]. Loss of *PTEN* function is one of the most frequent genomic aberrations in endometrioid EC [59] and is observed in more than 90% of high-grade EC [1]. PTEN, as a tumor suppressor, functions in the maintenance of genome integrity by regulating RAD51 and CHK1 activity. PTEN negatively regulates the PAT pathway and is also involved in cross-talk with other signaling pathways, including the RMM and estrogen receptor (ER) pathways. Loss of PTEN function in EC cells leads to the accumulation of DNA double-stranded breaks, and hence sensitivity to PARPis through synthetic lethality. Thus, EC with *PTEN-deficien*cy exhibits the RME and PAT pathway hyperactivation in response to PARPis [13, 60–63]. This may be the cause of the modest ORR observed in multiple clinical trials administrating PARPis in combination with RME or PAT pathway inhibitors [24, 25]. Herein, it was observed that pBADS99 positively correlates with increased survival of *PTEN-deficient* EC cells. BAD serves as a common downstream mediator of RME and PAT signaling [37]. Targeting either RMM or PAT signaling alone or in combination with other drugs exhibits a limited response and such approaches, due to rescue kinases that also phosphorylate BAD [37], are often associated with therapy resistance and disease recurrence [40, 64]. Thus, directly targeting pBAD in combination with other drugs, such as PARPis in *PTEN-deficient* EC represents a rational strategy to avoid rescue mechanisms associated with MAPK and PI3K inhibition and improve prognosis (SI 12).

It was observed herein that the combined NPB-PARPi treatment completely ablated primary EC xenografts, whereas this approach did not fully ablate metastatic growth in 1/8 animals. Therefore, metastasized EC cells in this animal must have acquired resistance to pBADS99 inhibition by NPB concomitant with resistance to PARP inhibition. Apart from anatomical and pharmacological considerations, one possibility is a clonal selection of EC stem-like cells (ECSCs) with BAD phosphorylation independent survival mechanisms during treatment [65–67]. It should be noted that mouse models with *Bad* deletion or knock-in of *Bad* mutated on serine residues 112 and 136 are viable and largely phenotypically normal; hence phosphorylated BAD/BAD is dispensable [37]. BAD independent cellular survival could include the activation of alternative ECSC pathways, such as HEDGHOG, NOTCH1, and Wingless-INT (Wnt)/β-CATENIN which could provide mechanisms to overcome combined NPB-PARPi treatment. Indeed, small subsets of EC cells expressing ALDH1, CD44, CD55, CD117, and CD133 possess higher capacities for self-renewal, de-differentiation, and metastasis that subsequently promote mechanisms for resistance to therapy [67–70]. Also, another potential mechanism to overcome the loss of BAD phosphorylation is through the mitophagy process in cells to clear damaged mitochondria. PINK1-dependent or BNIP3/NIX-mediated mitophagy has been reported to promote chemoresistance in CSCs of various cancers [71]. Moreover, high expression of 53BP1 in metastasized cancer cells [72] may also hinder NPB-PARPi efficacy through an elevated capacity for resistance to PARPis [73, 74]. Thus, further development of more potent and NPB-based derivatives, in combination with PARPis, would assist to obviate such an undesirable outcome. Regardless, the combined inhibition of pBADS99 and PARP reported herein is far superior to the use of PARPis alone and offers a mechanistically rational therapeutic approach to improve outcomes in *PTEN*-deficient EC.

## Supplementary information

Supplementary Information

reproducibility checklist

## Data Availability

The datasets generated during and/or analyzed during the current study are available from the corresponding author on reasonable request.
