# Supplementary material for: Inhibition of BAD-Ser99 phosphorylation synergizes with PARP inhibition to ablate PTEN-deficient endometrial carcinoma
Source: Cell Death Dis. 2022 Jun 20;13(6):558. doi: 10.1038/s41419-022-04982-8 (PMC9209517; doi:10.1038/s41419-022-04982-8)
Supplement: Supplementary file 1 — Supplementary Information [file 41419_2022_4982_MOESM1_ESM.pdf]

Supplementary Information for

Inhibition of BAD-Ser99 phosphorylation synergizes with PARP inhibition to ablate *PTEN*-deficient endometrial carcinoma

Xi Zhang[#1, 2] Peng Huang[# 2], Liqiong Wang [3], Shu Chen [2], Basappa Basappa [4], Tao Zhu [5, 6], Peter E. Lobie[* 1, 2, 7] and Vijay Pandey [* 2, 7]

1. *Shenzhen Bay Laboratory, Shenzhen 518055, Guangdong, China*
2. *Tsinghua Berkeley Shenzhen Institute, Tsinghua Shenzhen International Graduate School, Tsinghua University, Shenzhen 518055, PR China*
3. *Department of Gynecology and Obstetrics, the University of Hong Kong-Shenzhen Hospital, Shenzhen 518053, Guangdong, China.*
4. *Department of Studies in Organic Chemistry, University of Mysore, Manasagangotri, 570006 Mysore, India*
5. *Department of Oncology of the First Affiliated Hospital, Division of Life Sciences and Medicine, University of Science and Technology of China, Hefei, Anhui 230027, China;*
6. *Hefei National Laboratory for Physical Sciences, the CAS Key Laboratory of Innate Immunity and Chronic Disease, Division of Life Sciences and Medicine, University of Science and Technology of China, Hefei, Anhui 230027, China;*
7. *Institute of Biopharmaceutical and Health Engineering, Tsinghua Shenzhen International Graduate School, Tsinghua University, Shenzhen 518055, PR China*

Corresponding authors:

Peter E. Lobie, Information Building, Tsinghua Shenzhen International Graduate School, Nanshan, Shenzhen, 518055 P.R. China, Email: plobie@sz.tsinghua.edu.cn

Vijay Pandey, Room1209, Information Building, Tsinghua Shenzhen International Graduate School, Nanshan, Shenzhen, 518055 P.R. China, Email: vijay.pandey@sz.tsinghua.edu.cn

[#] These authors contributed equally to this study.

Supplementary Information 1.

(A) PARP inhibitor monotherapy associated toxicities in endometrial carcinoma.

**NCT02506816: Preoperative Olaparib Endometrial Carcinoma Study (POLEN) (n=36)**	
Adverse event	Affected/ at Risk (%)
Post-surgical wound infection	1/36 (2.75%)
Neutropenia	10/36 (27.78%)
Anemia	9/36 (25.00%)
Nausea	15/36 (41.67%)
Vomiting	6/36 (16/67%)
Diarrhea	5/36 (13.89%)
Asthenia	9/36 (25.00%)

(B) Ongoing Clinical Trials of Combined Inhibition of PARP and RAS/MEK/ERK or PI3K/AKT/mTOR in EC.

NCT No.	PARPi	Combining Inhibitor	Inhibiting Target	Country	Sponsor
NCT04586335	Olaparib	CYH33	PI3K	China	HaiheBiopharma Co., Ltd.
NCT03586661	Niraparib	Copanlisib	PI3K	USA	M.D. Anderson Cancer Center
NCT02208375	Olaparib	Vistusertib	mTOR	USA	M.D. Anderson Cancer Center
NCT02208375	Olaparib	Capivasertib	AKT	USA	M.D. Anderson Cancer Center
NCT02576444	Olaparib	Capivasertib	AKT	USA	Joseph Paul Eder

Supplementary Information 2.

A. Clinical pathology and molecular profiling of endometrial carcinoma cell lines utilized in the study.

Cell line	Clinical pathology					Molecular profiling		culture conditions
	**Site**	**Disease**	**Cell type**	**Age**	**Ethnicity**	***PTEN* status**	**Other mutant cancer driver genes**	
**KLE**	P	AC	E	64	Cau	WT	*P53, FBXW7*	DMEM/F12,10% FBS,1%PS at 37 °C and 5% CO_2
**Ishikawa**	P	AC	E	39	Asian	p.VL317fs<br>p. E288fs	*P53, RNF43, FBXW10, INPPL1, ARID1A, BEND3, TIMM44, CARD10, XIRP2, MTG1, MTSS1L, RB1, TUBE1, PUS7, ATF71P, BRAF, FUBP3, ARHGAP5, SDAD1, IPO11, FOLH1*	RPMI 1640, 10% FBS,1%PS at 37 °C and 5% CO_2
**RL95-2**	P	ASC	E	65	Cau	p.R173H<br>p. T321fs<br>p. K322fs<br>p.M134I	*P53, KMT2D, FAT2, ARID1A, ZFHX3, PI3KR1, ASPM, ATM, INPPL1, ZNRF3, BRAF, MGA, NF2, CDKN1B, BMPR2, HRAS, SOS1*	DMEM, 10% FBS,1%PS at 37 °C and 5% CO_2
**AN3CA**	RM	AC	E	55	Cau	p. R130fs	*P53, FBXW7, ZFHX3, CCAR1, LARP4B, ARFGEF1, ARFGEF2, MAP3K4, SETD2, NSD1, CTCF, LNPEP, AKAP9, MYH9, ELF3, SRGAP1, ZNF292, AHNAK, BAZ2B, MECOM*	DMEM, 10% FBS,1%PS at 37 °C and 5% CO_2S

Note: EC, endometrial cancer; P, Primary; RM, Recurrent metastasis; AC, Adenocarcinoma; ASC, Adenosquamous; E, epithelial; Cau, Caucasian; A, Asian; PS: penicillin/streptomycin

Note: Whereas cells were maintained and expanded with 10% FBS in the respective media, all experiments herein were performed with 2% FBS in the respective media.

B. **Quantitative PCR Analysis:** Total cellular RNA was extracted and converted to cDNA by use of Hifair® III 1st Standard cDNA Synthesis Kit (gDNA digester plus) (YEASEN.Inc, Shanghai, PR China) as per manufacturer's instructions. Quantitative PCR (qPCR) was performed using an CFX96 real-time PCR system (BIO-RAD, CA, USA). Briefly, multiple gene markers and an endogenous housekeeping gene (β-ACTIN) were used for real-time PCR analysis using the Hieff qPCR SYBR Green Master Mix (YEASEN.Inc, Shanghai, PR China). Each marker was determined in triplicate in a 96-

well plate using a 2-step amplification program of initial denaturation at 95℃ for 5 min, followed by 40 cycles of 95℃ for 10s and 60℃ for 30s. At the end of the amplification, a melting curve analysis was performed, consisting of denaturation at 95℃ for 1 min and re-annealing at 55℃ for 1 min. Standard curves were generated from each experimental plate using serial 5-fold dilutions of untreated cDNA. The geometric mean of the cycle threshold (Ct) value for each reaction was calculated. The changes of gene expression were expressed as fold change and represented by the average of 3 experiments ($P < 0.05$). Primer sequence: *PTEN-F: 5'-CAGGGCAGTGATTGACTACAG-3'*, *PTEN-R: 5'-GATTGACAAGCCCGAAGATTTC-3'*.

sgRNA Sequences targeting *PTEN* (GENEWIZ, Suzhou, China) cloned into the *pSpCas9(BB)-2A-Puro (PX459) V2.0* construct (#62988, Addgene, Cambridge, MA, USA) were list as followed:

#1 sgRNA	5′-*GGAACCTGGAGCGGGGGGGAGAAGCGTTCC*-3′
#2 sgRNA	5′-*GGAACTCAGACTTTTGTAATTTGTGGTTCC*-3′
#3 sgRNA	5′-*GGAACTTATCCAAACATTATTGCTAGTTCC*-3′
#4 sgRNA	5′-*GGAACCCTACCTCTGCAATTAAATTGTTCC*-3′

C. Western blot: Western blot analysis was performed as previously described (1-3). Briefly, cells were lysed in RIPA buffer and the cell lysate were resolved using SDS polyacrylamide gel electrophoresis and visualized with Clarity™ and Clarity Max™ Western ECL Blotting Substrates (BIO-RAD, USA). The secondary anti-rabbit, anti-mouse and anti-goat horseradish peroxidase (HRP)-conjugated antibodies were obtained from Cell Signaling Technology, USA.

The list of primary and secondary antibodies used in this study.

**Protein marker**	**Company/catalog number**
Phospho-BAD (Ser136)	Cell Signaling D25H8
Phospho-BAD (Ser112)	Cell Signaling 40A9
BAD (11E3)	Cell Signaling 9268
Phospho-BAD (Ser136) (IHC)	GeneTex GTX79125
BAD (IHC)	GeneTex GTX50417
β-ACTIN	Santa Cruz sc-47778
CHK1	Cell Signaling 2G1D5
phospho-CHK1 (Ser345)	Cell Signaling 133D3
Caspase-3	Santa Cruz sc-7272

Caspase-7	Santa Cruz sc-56063
phospho-Histone H2A.X (Ser139)	Cell Signaling 2577S
RAD51	Abcam ab133534
ALDHA1	Cell Signaling 36671
CD44	Abcam ab157107
PI3 Kinase p85	Cell Signaling 13666
PI3 Kinase p110	Cell Signaling 4249
Akt	Cell Signaling 4685
Phospho-Akt(Tyr308)	Cell Signaling 4056
Phospho-Akt(Ser473)	Cell Signaling 4060
PARP	Cell Signaling 9532
PTEN	Abcam ab170941
Phospho-PTEN(Ser380)	Cell Signaling 9551
IgG H&L (Alexa Fluor® 488)	Abcam ab150077
IgG H&L (Alexa Fluor® 647)	Abcam ab150079

D. *Xenograft* studies were performed according to guidelines for the care and use of laboratory animals, approved by the Laboratory Animal Ethics Committee (Certificate number: YW) at Peking University, Shenzhen as previously described (4), and ethical approval obtained from Tsinghua Shenzhen International Graduate School (Number:9, Year 2020). Briefly, AN3CA-GFP cells (5×10^6 cells) were injected subcutaneously (s.c.) into the right flanks of five-week-old female BALB/c nude mice. Mice (n=8) bearing similar xenograft size were randomly assigned to different treatment arms: control, NPB, Olaparib, or NPB-Olaparib as summarized in SI 9. AN3CA-GFP xenograft volume was determined by calipers and the subcutaneous fluorescence area of AN3CA-GFP xenografts was observed by a fluorescence imaging system (IVIS Spectrum, PerkinElmer, US) in all the implanted mice before sacrificing (Figure 4B). After all the mice were sacrificed, organs including lung, liver, and lymph nodes were dissected and removed for further examination using fluorescence imaging (IVIS Spectrum, Bruker, US).

E. **Patient-derived cancer cell (PDC) and organoid (PDO) culture and treatment**: Specimens used for PDC and PDO in this study were obtained from HKU-SZH. Fresh human EC tissues were obtained with written informed consent and approval from the patients and the Ethical Committee of the University of Hong Kong-Shenzhen Hospital (HKU-SZH, research No. hkuszh2019105, approval No.伦 [2019]096, Date: 2019.03.26). Tumor tissues were diagnosed pathologically. The detailed information of the patient is listed in SI 11A. Tumor tissue was minced into tiny pieces with scissors, and the tissue fragments were incubated in serum-free Advanced Dulbecco's modified Eagle's medium

(DMEM)/F12 (Thermo Fisher Scientific, Inc., Waltham, MA, USA) containing 1.5 mg/ml collagenase IV (Gibco; Thermo Fisher Scientific, Inc.) and 1% penicillin/streptomycin (Thermo Fisher Scientific, Inc.) at 37 ℃ for 2-4h with continuous slight shaking. The cells were filtered through a cell strainer after digestion followed by centrifugation for 5 min at a speed of 500 r/min. The supernatant was removed and the pellets were washed with phosphate-buffered saline (PBS), and centrifuged as described above. Single primary cells were resuspended in Advanced Dulbecco's modified Eagle's medium (DMEM)/F12 for use in subsequent organoid culture. For organoid culture, Matrigel (354262, Corning, US) was added to the plate and polymerized for 15 min at 37 ℃, and then growth medium mixed primary cells supplemented with 2% FBS, 50% Matrigel (354262, Corning, USA), 1x N2 Supplement (Gbico; Thermo Fisher Scientific, Inc.), 1x B27 (Gbico; Thermo Fisher Scientific, Inc.), 50ng/ml recombinant human EGF (PEPROTECH; USA), and 20 ng/ml basic fibroblast growth factor (Sigma-Aldrich; Merck). 1.25μM N-Acetylcysteine (Sigma-Aldrich; Merck), Rock inhibitor (Y-27632) (Sigma-Aldrich; Merck), 10nM 17-β Estradiol (Sigma-Aldrich; Merck) and A83-01 (Sigma-Aldrich; Merck) were added to the wells (200μl/well in 48-well plates and 500μl /well in 24-well plates). PDO usually formed within 3 days of primary culture and were expanded for 9 days. Treatment was begun on the third day after organoid formation and medium with treatment was changed every 2 days. Organoids with diameters >100 μm were counted under an inverted microscope to determine organoid expansion. At termination, viability and apoptosis using the ApoTox-Glo Triplex Assay Kit (G6320, Promega, China) was determined (5-9).

F. The construction of *Flag-h*BAD-WT, and -S99A and transient transfection: Nucleotide mutations from TCG into GCC were introduced to the coding sequence (CDS) so as to achieve *BAD-S99A* in which Ser99 was replaced with Ala. Flag-tag encoding DNA sequence (5-ATGGATTACAAGGACGACGATGACAAGGGCAGC-3) was added at the 5' of *hBAD-WT* and *hBAD-S99A CDSs* so as to monitor the expression of exogenous BAD protein in cells. These *flag-hBAD* related cDNAs were then sub-cloned into pcDNA3.1(+) plasmid vector (with Nhe I (R0131, New England Biolabs (Beijing) Ltd., PRC) and EcoR I（R0101, New England Biolabs (Beijing) Ltd., PRC）restriction endonuclease. The CDSs of *flag-hBAD-WT and -S99A* were sequenced by Sangon Biotech (Shanghai, PRC) and are listed in the following table. Constructs were designated as pcDNA3.1-flag-*h*BAD-WT and pcDNA3.1-flag-*h*BAD-S99A. Cells were transiently transfected using FuGENE® 6 Transfection Reagent (E2692, Promega, Madison, WI, USA) as per manufacturer's instructions and previously described (1).

CDSs of Flag-hBAD-WT and -S99A

cDNA	Coding sequence (CDS)
hBAD-WT	5'**ATGGATTACAAGGACGACGATGACAAGGGCAGC**ATGTTCCAGATCCCAGAGTTTGAGCCGAGTG<br>AGCAGGAAGACTCCAGCTCTGCAGAGAGGGGCCTGGGCCCCAGCCCCGCAGGGGACGGGCCCTCAG<br>GCTCCGGCAAGCATCATCGCCAGGCCCCAGGCCTCCTGTGGGACGCCAGTCACCAGCAGGAGCAGCC<br>AACCAGCAGCAGCCATCATGGAGGCGCTGGGGCTGTGGAGATCCGGAGTCGCCACAGCTCCTACCCCG<br>CGGGGACGGAGGACGACGAAGGGATGGGGGAGGAGCCCAGCCCCTTTCGGGGCCGCTCGCGCTCGG<br>CGCCCCCCAACCTCTGGGCAGCACAGCGCTATGGCCGCGAGCTCCGGAGGATGAGTGACGAGTTTGTG<br>GACTCCTTTAAGAAGGGACTTCCTCGCCCGAAGAGCGCGGGCACAGCAACGCAGATGCGGCAAAGCTC<br>CAGCTGGACGCGAGTCTTCCAGTCCTGGTGGGATCGGAACTTGGGCAGGGGAAGCTCCGCCCCCTCCC<br>AGTGA-3'
hBAD-S99A	5'**ATGGATTACAAGGACGACGATGACAAGGGCAGC**ATGTTCCAGATCCCAGAGTTTGAGCCGAGTG<br>AGCAGGAAGACTCCAGCTCTGCAGAGAGGGGCCTGGGCCCCAGCCCCGCAGGGGACGGGCCCTCAG<br>GCTCCGGCAAGCATCATCGCCAGGCCCCAGGCCTCCTGTGGGACGCCAGTCACCAGCAGGAGCAGCC<br>AACCAGCAGCAGCCATCATGGAGGCGCTGGGGCTGTGGAGATCCGGAGTCGCCACAGCTCCTACCCCG<br>CGGGGACGGAGGACGACGAAGGGATGGGGGAGGAGCCCAGCCCCTTTCGGGGCCGCTCGCGC**GCC**G<br>CGCCCCCCAACCTCTGGGCAGCACAGCGCTATGGCCGCGAGCTCCGGAGGATGAGTGACGAGTTTGTG<br>GACTCCTTTAAGAAGGGACTTCCTCGCCCGAAGAGCGCGGGCACAGCAACGCAGATGCGGCAAAGCTC<br>CAGCTGGACGCGAGTCTTCCAGTCCTGGTGGGATCGGAACTTGGGCAGGGGAAGCTCCGCCCCCTCCC<br>AGTGA-3'

Supplementary information 3

A. *hBADS99A-Flag* transfection

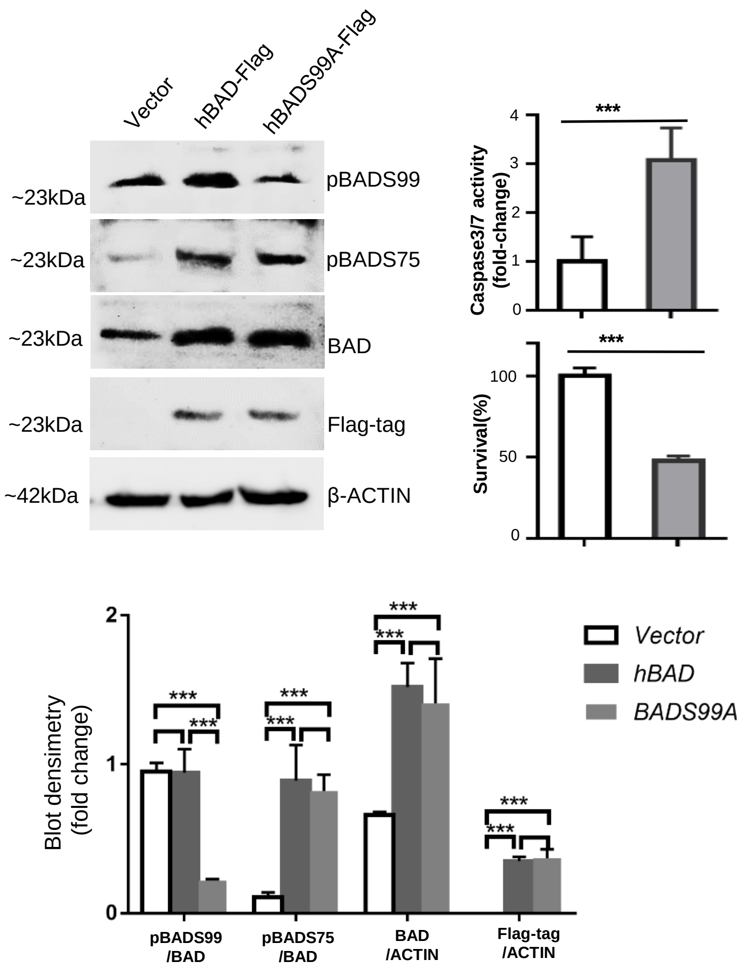

B. Western Blot analysis

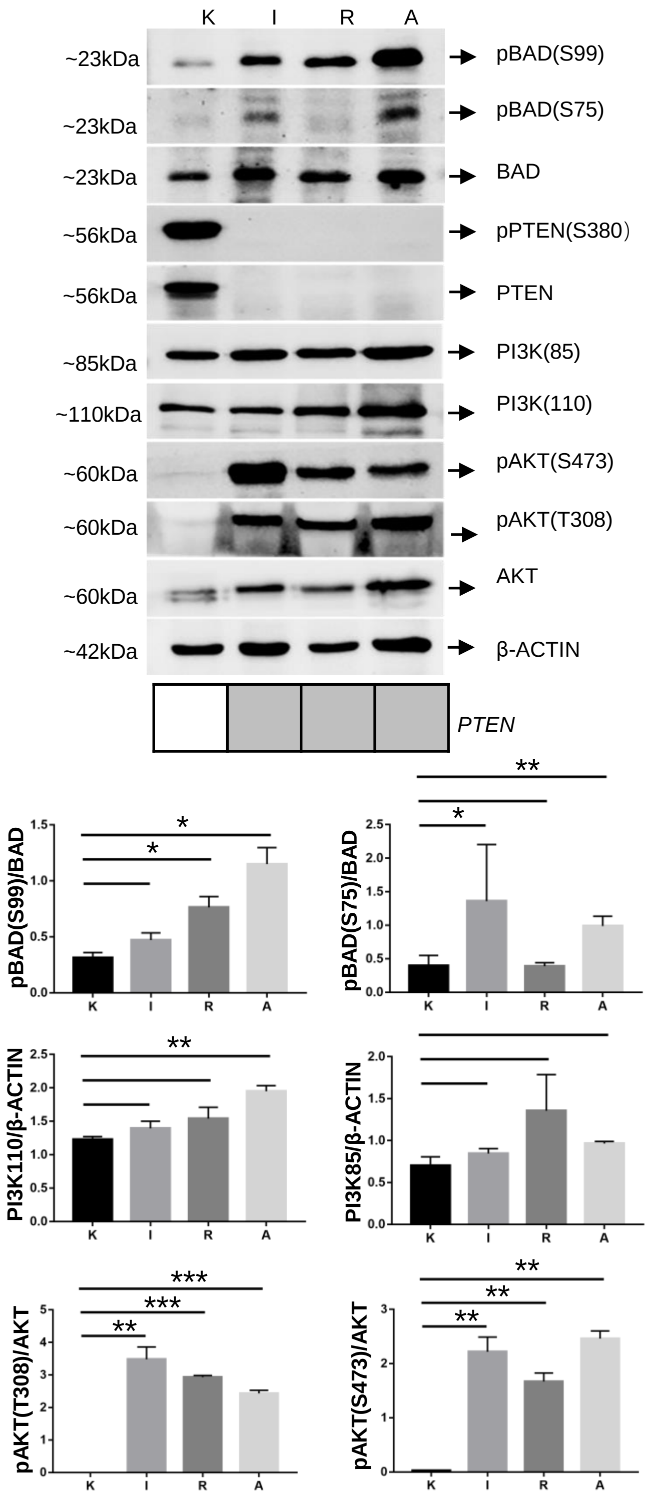

Supplementary Information 3

A. Up-left: Forced expression of Flag-hBAD or Flag-hBADS99A in AN3CA was confirmed by Western Blot analysis. The sizes of detected protein bands in kDa are shown on the left side, and the tag of each band was indicated by arrow on the right side. Up-right: cell viability and caspase3/7 activity were evaluated using the ApoTox-Glo Triplex Assay Kit in AN3CA cells after transfection (n=3). Below: quantification of western blot. Statistical significance was assessed by using an unpaired two-tailed Student's t test ($P<0.05$ was considered as significant) using GraphPad Prism.

B. Western blot analysis was used to assess the levels of PTEN as well as phosphorylated PTEN and PI3K, AKT, phosphorylated AKT, pBADS75/99 and BAD, determined in 4 endometroid endometrial carcinoma (EC) cell lines. Soluble whole cell extracts were run on an SDS-PAGE and immunoblotted as described in materials and methods. β-ACTIN was used as input control for cell lysate. The sizes of detected protein bands in kDa are shown on the left side, and the tag of each band is indicated by arrow on the right side. Densitometries of protein bands were determined using ImageJ software (https://imagej.nih.gov/ij/). Grids: Specific *PTEN* mutations in the EC cell lines are indicated by the grey colored square. Note: KLE=K, Ishikawa=I, RL95-2=R, AN3CA=A. Below: Quantification of western blot. Statistical significance was assessed by using an unpaired two-tailed Student's t test ($P<0.05$ was considered as significant) using GraphPad Prism.

Points are mean of triplicate experiments; bars, ±SD. *** $p < 0.001$.

Supplementary Information 4.

(A) IC_{50} was calculated in 4 EC cell lines treated with the indicated concentration of NPB and PARP inhibitor for 6 days.

Cell lines	IC_{50} ±SD (μM)						
Drug	NPB	OLA	OLA+ NPB(1μM)	RUC	RUC+ NPB(1μM)	TAL	TAL+ NPB(1μM)
Relative PARP-trapping capacity		PARP1+++ PARP2++++		PARP++++		PARP1+++	
KLE	0.68±0.03	1.3±0.003	0.006±0.0018	1.007±0.012	0.012±0.0014	0.209±0.006	0.0057±0.0002
Ishikawa	0.66±0.3	0.085±0.02	0.004±0.0001	0.59±0.001	0.0364±0.001	0.087±0.001	0.01±0.003
RL95-2	1.66±0.02	0.79±0.01	0.013±0.003	0.11±0.009	0.024±0.013	0.155±0.009	0.0225±0.001
AN3CA	0.66±0.02	0.13±0.006	0.004±0.001	0.819±0.002	0.053±0.006	0.127±0.003	0.015±0.007

Data are represented as mean ± SD.

(B) Combination Index (CI) of NPB and PARP inhibitor combination therapy were calculated in 4 cell models in constant ratio concentration (10-fold serial dilution).

Cell lines	Doses																	
	NPB/OLA						NPB/RUC						NPB/TAL					
	10^{-3}	10^{-2}	10^{-1}	10^{0}	10^{1}	10^{2}	10^{-3}	10^{-2}	10^{-1}	10^{0}	10^{1}	10^{2}	10^{-3}	10^{-2}	10^{-1}	10^{0}	10^{1}	10^{2}
KLE	0.0374	0.0174	0.0087	0.0025	0.0020	0.0010	0.0116	0.0244	0.0229	0.1140	0.0092	0.0100	0.0583	0.0219	0.0151	0.0065	0.0240	0.2400
Ishikawa	0.01267	0.0123	0.0316	0.0699	0.0597	0.0066	0.0122	0.0208	0.0241	0.1109	0.0689	0.0584	0.0073	0.0191	0.0530	0.0893	0.0689	0.0584
RL95-2	0.1506	0.3267	0.0554	0.0560	0.0728	0.1168	0.0772	0.1126	0.0286	0.0952	0.1453	0.0858	0.0405	0.0918	0.0266	0.0145	0.0087	0.0872
AN3CA	0.0222	0.0115	0.0064	0.009	0.0006	0.0001	0.1637	0.1500	0.0974	0.0540	0.0092	0.0167	0.2228	0.2082	0.0427	0.0092	0.0038	0.0172

Supplementary information 5

A. Flow cytometry

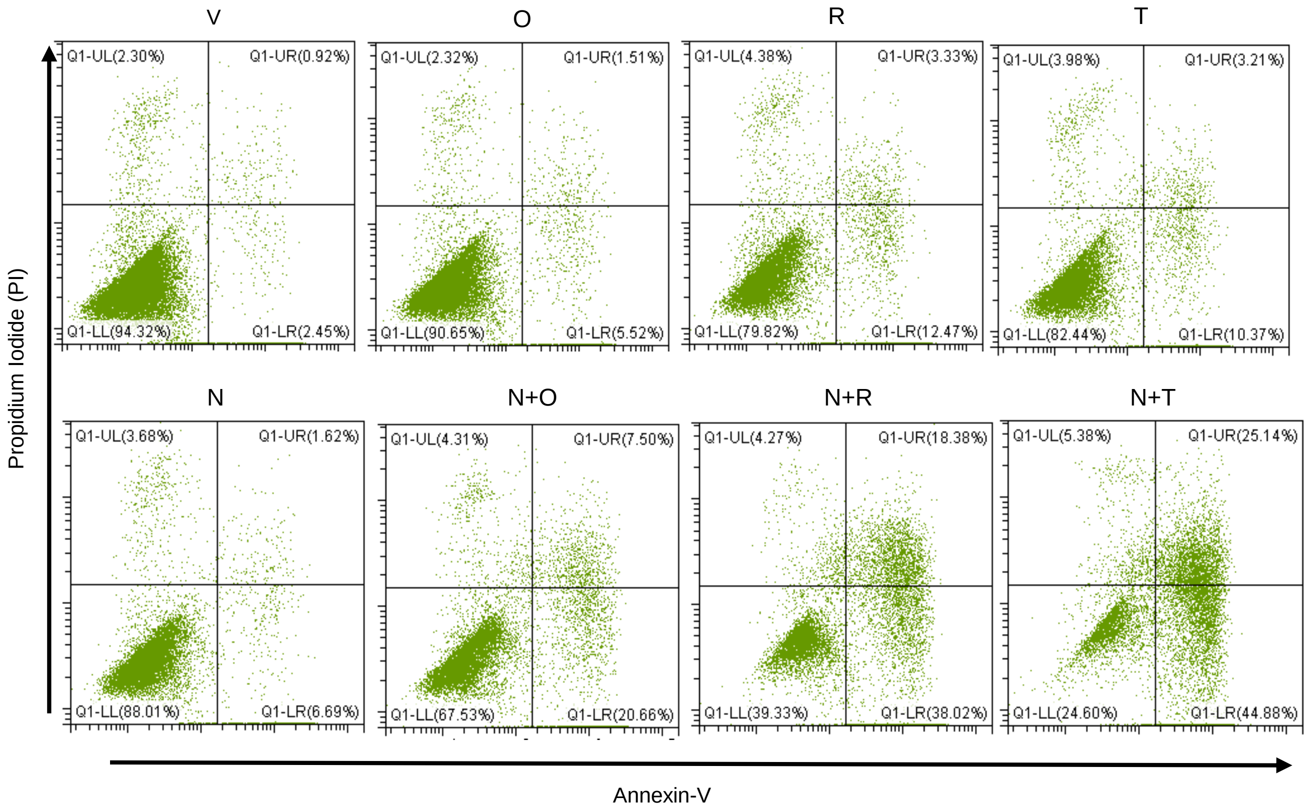

B. Cell cycle analysis

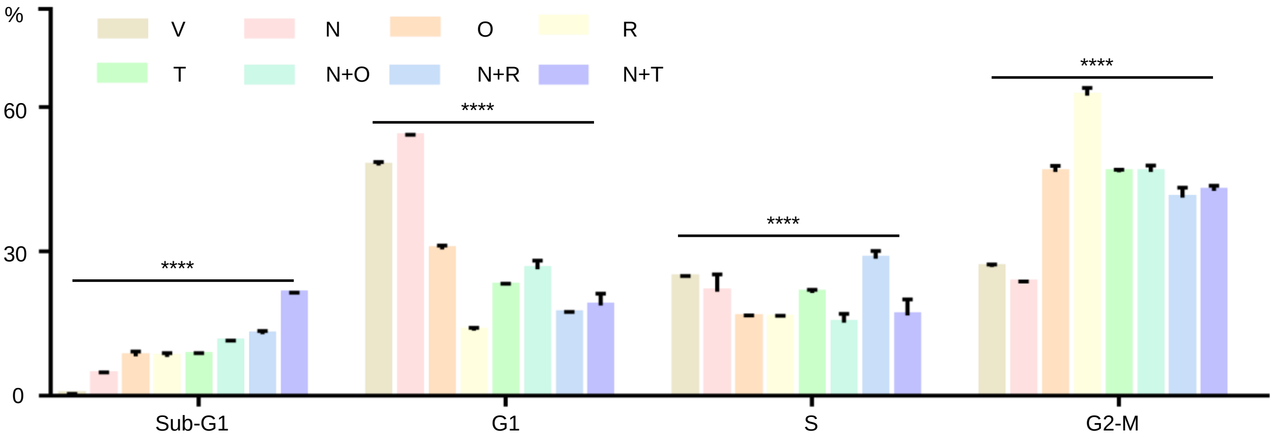

Supplementary Information 5.

(A) Representative flow cytometry plots using Annexin V-FITC/PI staining for apoptotic cell death of AN3CA cells measured after treatment with NPB (N), Olaparib (O), Rucaparib (R), and Talazoparib (T) or combinations using flow cytometry analysis at 72 hours as described in materials and methods (n=3).

(B) Sub-population of cells in cell cycle phase: G1, S, and G2-M phase after 72 hours of treatment by NPB (N), Olaparib (O), Rucaparib (R), and Talazoparib (T) or combinations (n=3).

Supplementary information 6

Densitometry analysis

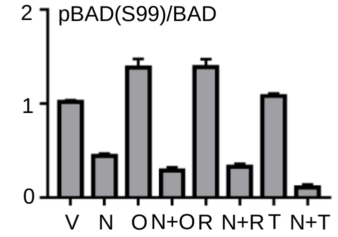

p-value of t-test in pBAD(S99)/BAD							
	N	O	R	T	N+O	N+R	N+T
V	**0.0019****	**0.0310***	**0.0261***	0.1215	**0.0016****	**0.0013****	**0.0009*****
N		**0.0051****	**0.0044****	**0.0019****	**0.0401***	0.0602	**0.0079****
O			0.9601	**0.0463***	**0.0040****	**0.0041****	**0.0029****
R				**0.0392***	**0.0034****	**0.0035****	**0.0025****
T					**0.0016****	**0.0014****	**0.0009*****

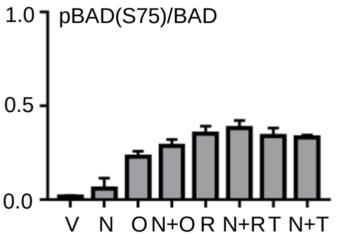

p-value of t-test in pBAD(S75)/BAD							
	N	O	R	T	N+O	N+R	N+T
V	0.3912	**0.0089****	**0.0081****	**0.0086****	**0.0087****	**0.0068****	**0.0011****
N		0.0599	**0.0274***	**0.0293***	**0.0395***	**0.0227***	**0.0214***
O			0.0796	0.0927	0.2280	0.0532	**0.0465***
R				0.8356	0.2379	0.5528	0.5918
T					0.2943	0.4453	0.7818

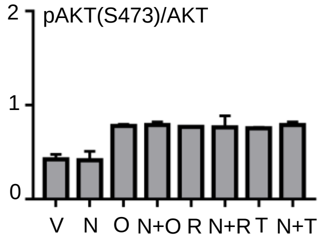

p-value of t-test in pAKT(S473)/AKT							
	N	O	R	T	N+O	N+R	N+T
V	0.9126	**0.0142***	**0.0134***	**0.0153***	**0.0163***	0.0746	**0.0163***
N		**0.0364***	**0.0369***	**0.0406***	**0.0371***	0.0918	**0.0371***
O			0.5918	0.2999	0.7643	0.8845	0.7643
R				0.3118	0.5149	0.9608	0.5149
T					0.3233	0.9222	0.3233

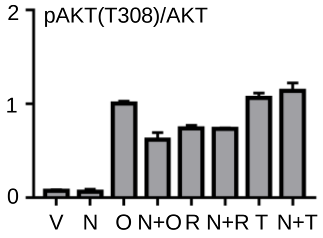

p-value of t-test in pAKT(T308)/AKT							
	N	O	R	T	N+O	N+R	N+T
V	0.6985	**0.0006*****	**0.0016****	**0.0017****	**0.0104***	**0.0005*****	**0.0032****
N		**0.0009*****	**0.0022****	**0.0020****	**0.0109***	**0.0011****	**0.0034****
O			**0.0143***	0.3118	**0.0223***	**0.0068****	0.1573
R				**0.0204***	0.1854	0.8698	**0.0248***
T					**0.0226***	**0.0153***	0.3828

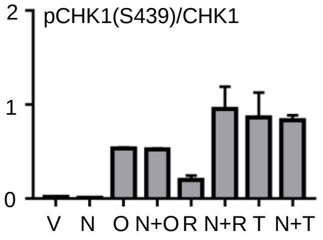

p-value of t-test in pCHK1(S439)/CHK1							
	N	O	R	T	N+O	N+R	N+T
V	0.2951	**0.0005*****	**0.0365***	**0.0471***	**0.0005*****	**0.0315***	**0.0024****
N		**0.0004*****	**0.0330***	**0.0461***	**0.0004*****	**0.0309***	**0.0024****
O			**0.0116***	0.2250	0.5528	0.1325	**0.0184***
R				0.0750	**0.0123***	**0.0490***	**0.0069****
T					0.2158	0.7578	0.8914

Supplementary Information 6.

Left: Western blot analysis was used to assess the level of various protein markers and protein activity in AN3CA cells after treatment with NPB (N), Olaparib (O), Rucaparib (R), and Talazoparib (T) or combinations. Soluble whole-cell extracts were run on an SDS-PAGE and immunoblotted as described in materials and methods. β-ACTIN was used as input control for cell lysate. Densitometries of protein bands were subsequently determined using ImageJ software (https://imagej.nih.gov/ij/). Right: Differences in relative protein marker quantification values between each of the two AN3CA treatment groups were assessed via t-test analysis. P-values of the t-test were indicated in the table. Statistical significance was assessed by using an unpaired two-tailed Student's t-test ($P<0.05$ was considered significant) using GraphPad Prism.

Columns are mean of triplicate experiments; bars, ±SD. * $p <0.05$, ** $p <0.01$, *** $p <0.001$, **** $p <0.0001$

Supplementary information 7

Densitometry analysis

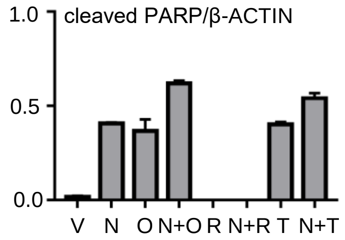

p-value of t-test in PARP/β-ACTIN							
	N	O	R	T	N+O	N+R	N+T
V	0.0003***	0.0163*	0.0955	0.0008***	0.0003***	0.0955	0.0015**
N		0.4702	0.0002***	0.6985	0.0027**	0.0002***	0.0225*
O			0.0149*	0.5270	0.0312*	0.0149*	0.0709
R				0.0006***	0.0003***	N/A	0.0014**
T					0.0041**	0.0006***	0.0246*

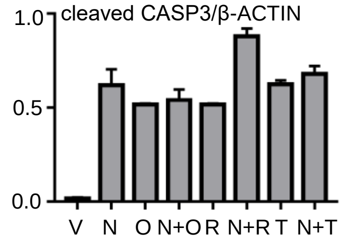

p-value of t-test in cleaved CASP3/β-ACTIN							
	N	O	R	T	N+O	N+R	N+T
V	0.0098**	0.0002***	0.0002***	0.0007***	0.0058**	0.0012**	0.0021**
N		0.2233	0.2233	0.9429	0.3828	0.0606	0.4655
O			>0.9999	0.0200*	0.5984	0.0069**	0.0323*
R				0.0200*	0.5984	0.0069**	0.0323*
T					0.1849	0.0169*	0.2427

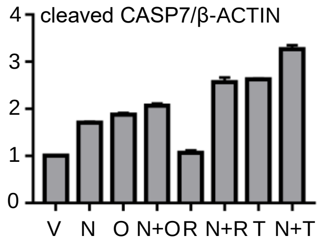

p-value of t-test in cleaved CASP7/β-ACTIN							
	N	O	R	T	N+O	N+R	N+T
V	0.0008***	0.0012**	0.2724	<0.0001****	0.0011***	0.0020**	0.0007***
N		0.0422*	0.0048**	0.0007***	0.0120*	0.0069**	0.0016**
O			0.0038**	0.0020**	0.0516	0.0116*	0.0023**
R				0.0007***	0.0028**	0.0028**	0.0011**
T					0.0046**	0.5226	0.0091**

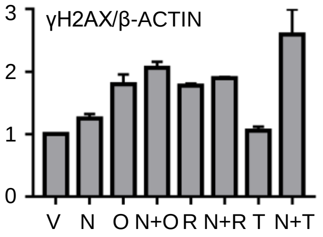

p-value of t-test in γH2AX/β-ACTIN							
	N	O	R	T	N+O	N+R	N+T
V	0.0377*	0.0184*	0.0007***	0.4226	0.0037**	0.0001***	0.0305*
N		0.0450*	0.0102*	0.1056	0.0100**	0.0061**	0.0433*
O			0.8745	0.0250*	0.1738	0.4608	0.1214
R				0.0054**	0.0525	0.0330*	0.1041
T					0.0065**	0.0036**	0.0333*

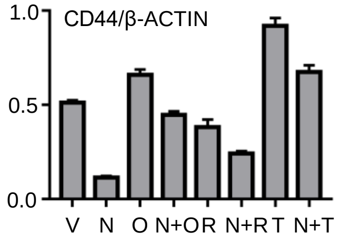

p-value of t-test in CD44/β-ACTIN							
	N	O	R	T	N+O	N+R	N+T
V	0.0008***	0.0215*	0.0544	0.0059**	0.0691	0.0027**	0.0256*
N		0.0014**	0.0129*	0.0014**	0.0023**	0.0079**	0.0021**
O			0.0162*	0.0187*	0.0133*	0.0028**	0.6855
R				0.0061**	0.1922	0.0474*	0.0171*
T					0.0049**	0.0022**	0.0245*

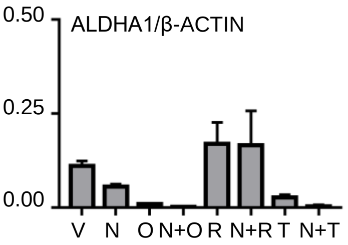

p-value of t-test in ALDHA1/β-ACTIN							
	N	O	R	T	N+O	N+R	N+T
V	0.0389*	0.0098**	0.2829	0.0182*	0.0086**	0.4910	0.0092**
N		0.0120*	0.1040	0.0618	0.0091**	0.2336	0.0114*
O			0.0569	0.0913	0.0207*	0.1392	0.1353
R				0.0709	0.0524	0.9537	0.0534
T					0.0487*	0.1677	0.0622

Supplementary Information 7.

Left: Western blot analysis was used to assess the level of various protein markers and protein activity in AN3CA cells after treatment with NPB (N), Olaparib (O), Rucaparib (R), and Talazoparib (T) or combinations. Soluble whole-cell extracts were run on an SDS-PAGE and immunoblotted as described in materials and methods. β-ACTIN was used as input control for cell lysate. Densitometries of protein bands were subsequently determined using ImageJ software (https://imagej.nih.gov/ij/). Right: Differences in relative protein marker quantification values between each of the two AN3CA treatment groups were assessed via t-test analysis. P-values of the t-test were indicated in the table. Statistical significance was assessed by using an unpaired two-tailed Student's t-test ($P<0.05$ was considered significant) using GraphPad Prism.

Columns are mean of triplicate experiments; bars, ±SD. * $p <0.05$, ** $p <0.01$, *** $p <0.001$, **** $p <0.0001$

Supplementary information 8

Scheme of HR-Reporter Assay

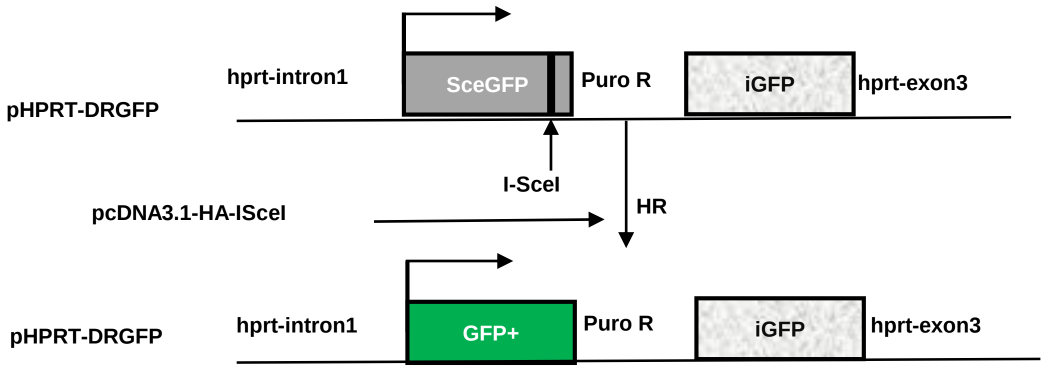

Supplementary Information 8.

Scheme of *HR-Reporter* Assay: The *HR-GFP reporter* consists of two copies of a mutant *GFP* gene. Cells were transfected with I-SceI expressing vector to induce DSB and which may be repaired by HR using *iGFP* as a template and restoring a functional *GFP* gene.

Supplementary information 9

A. KLE-KO cell line

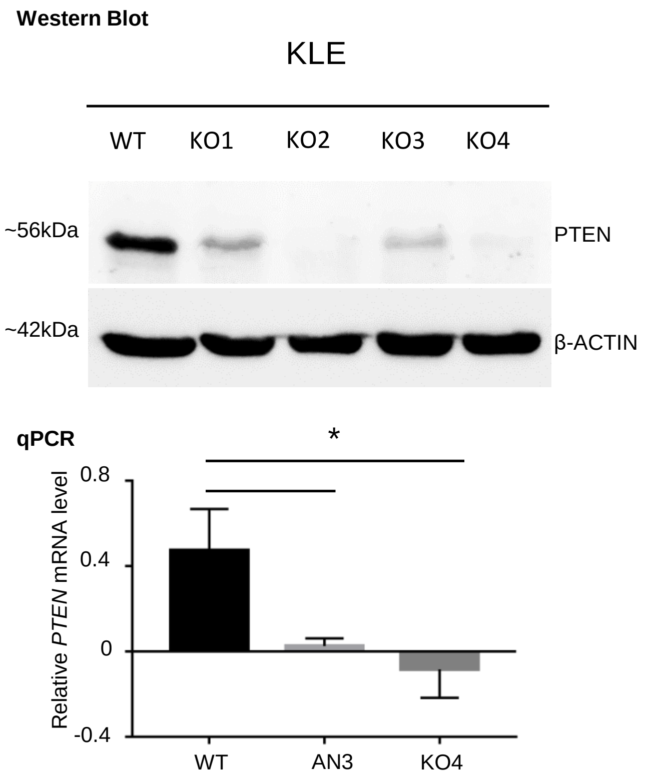

B. Densitometry analysis

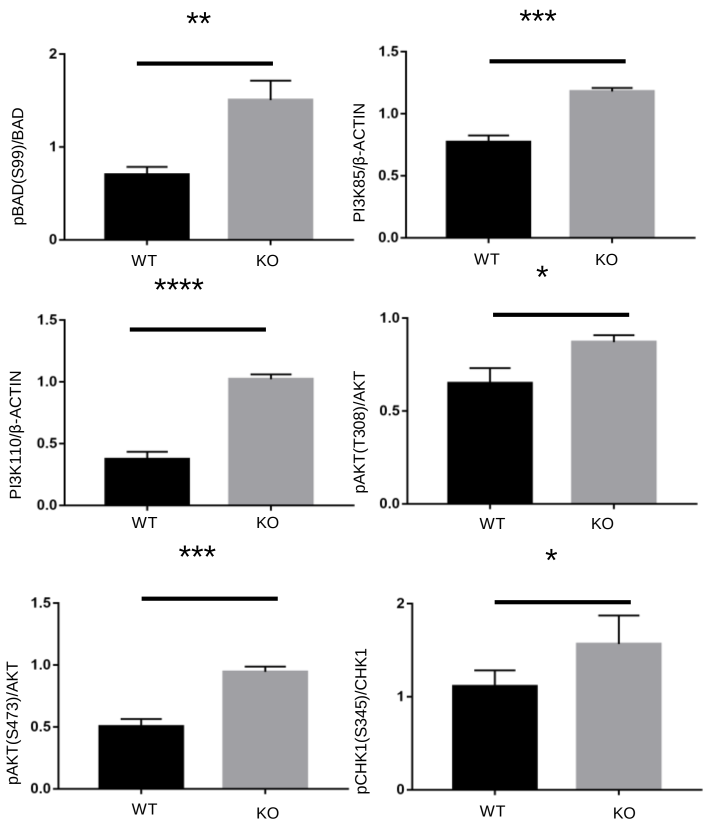

C. Fold change of NPB combined PARP inhibitors response in KLE-WT/KO cells

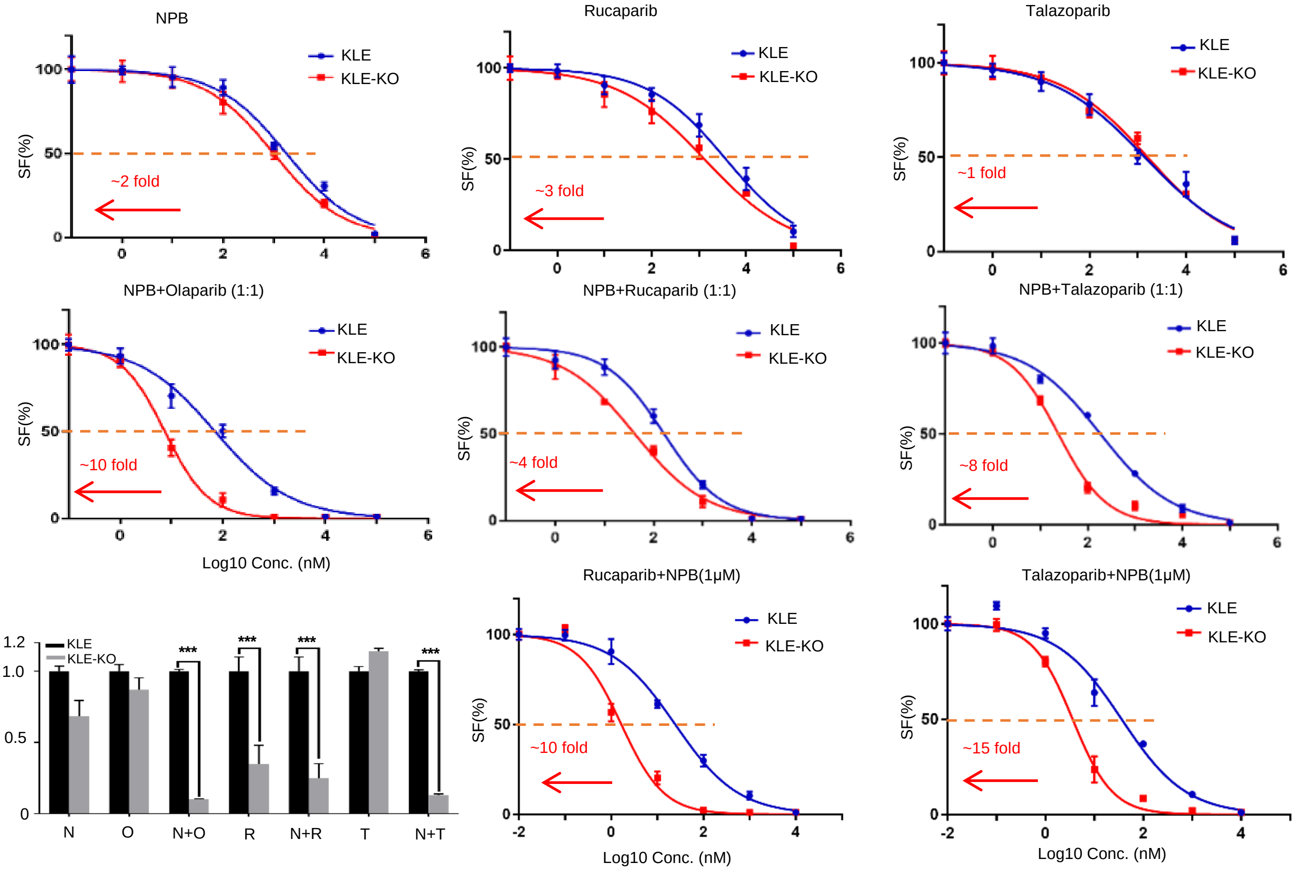

Supplementary Information 9.

(A) Up: Western blot analysis was used to assess the protein levels of PTEN in 4 KLE *PTEN-KO* cell lines. Soluble whole-cell extracts were run on an SDS-PAGE and immunoblotted as described in materials and methods. β-ACTIN was used as input control for cell lysate. The sizes of detected protein bands in kDa are shown on the left side, and the tag of each band was indicated on the right side. Below: RT-qPCR analysis was used to assess the mRNA levels of *PTEN* in 4 KLE *PTEN-KO* cell lines. Statistical significance was assessed by using an unpaired two-tailed Student's t-test ($P<0.05$ was considered significant) using GraphPad Prism.

(B) Quantification of the western blot in Figure 4A. Statistical significance was assessed by using an unpaired two-tailed Student's t-test ($P<0.05$ was considered significant) using GraphPad Prism.

(C) Cells were treated with the indicated concentration ($\log_{10}$ scale) of NPB and indicated PARP inhibitors for 6 days. The percentage of survival fraction was assessed using total cell number. Statistical significance was assessed by using an unpaired two-tailed Student's t-test ($P<0.05$ was considered significant) using GraphPad Prism.

Columns are mean of triplicate experiments; bars, ±SD. * $p < 0.05$, ** $p < 0.01$, *** $p < 0.001$, **** $p < 0.0001$

Supplementary information 10

Densitometry analysis

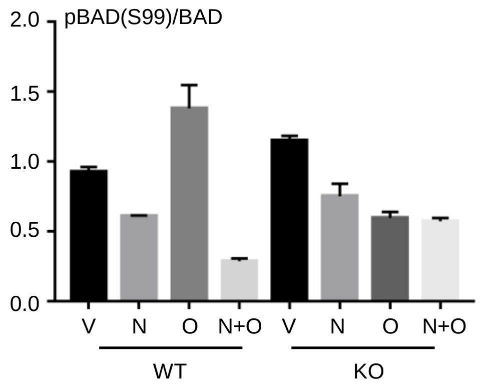

**p-value of t-test in pBAD(S99)/BAD**								
pBADS99/BAD		WT			KO			
		N	O	N+O	V	N	O	N+O
WT	V	**0.0062****	0.0633	**0.0020****	**0.0227***	0.1242	**0.0138***	**0.0070****
	N		**0.0225***	**0.0022****	**0.0020****	0.1550	0.7235	0.1689
	O			**0.0115***	0.1949	**0.0423***	**0.0232***	**0.0209***
	N+O				**0.0011****	**0.0191***	**0.0116***	**0.0059****
KO	V					**0.0278***	**0.0049****	**0.0026****
	N						0.1590	0.1126
	O							0.5596

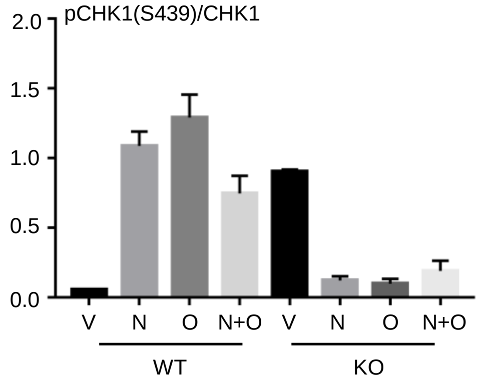

**p-value of t-test in pCHK1(S439)/CHK1**								
pBADS99/BAD		WT			KO			
		N	O	N+O	V	N	O	N+O
WT	V	**0.0049****	**0.0089****	**0.0164***	**0.0002*****	0.0844	0.2205	0.1201
	N		0.2810	0.0978	0.1276	**0.0061****	**0.0060****	**0.0098****
	O			0.0667	0.0816	**0.0103***	**0.0100***	**0.0134***
	N+O				0.2259	**0.0211***	**0.0201***	**0.0331***
KO	V					**0.0009*****	**0.0011****	**0.0055****
	N						0.5415	0.3456
	O							0.2504

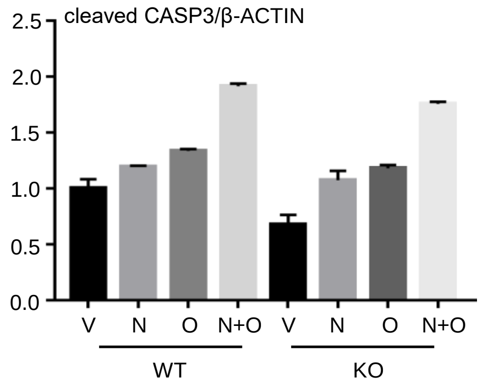

**p-value of t-test in cleaved CASP3/β-ACTIN**								
pBADS99/BAD		WT			KO			
		N	O	N+O	V	N	O	N+O
WT	V	0.0804	**0.0288***	**0.0042****	0.0614	0.4698	0.0993	**0.0060****
	N		**0.0087****	**0.0006*****	**0.0142***	0.1872	0.6523	**0.0008*****
	O			**0.0011****	**0.0089****	**0.0489***	**0.0230***	**0.0018****
	N+O				**0.0026****	**0.0053****	**0.0013****	**0.0165***
KO	V					**0.0436***	**0.0164***	**0.0034****
	N						0.2347	**0.0079****
	O							**0.0020****

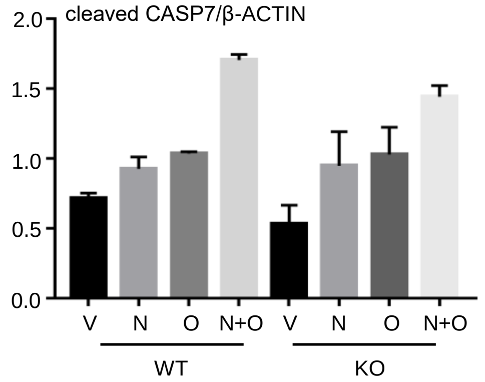

**p-value of t-test in cleaved CASP7/β-ACTIN**								
pBADS99/BAD		WT			KO			
		N	O	N+O	V	N	O	N+O
WT	V	0.0839	**0.0071****	**0.0015****	0.2016	0.3186	0.1546	**0.0074****
	N		0.2139	**0.0071****	0.0726	0.9216	0.5626	**0.0248***
	O			**0.0020****	**0.0344***	0.6603	0.9708	**0.0197***
	N+O				**0.0071****	**0.0495***	**0.0406***	0.0535
KO	V					0.1702	0.0968	**0.0146***
	N						0.7439	0.1132
	O							0.1098

Supplementary Information 10.

Left: Western blot analysis was used to assess the level of various protein markers and protein activity in KLE-WT and KLE-KO cells after treatment with NPB (N), Olaparib (O), or combinations. Soluble whole-cell extracts were run on an SDS-PAGE and immunoblotted as described in materials and methods. β-ACTIN was used as input control for cell lysate. Densitometries of protein bands were subsequently determined using ImageJ software (https://imagej.nih.gov/ij/). Right: Differences in relative protein marker quantification values between each two AN3CA treatment groups were assessed via t-test analysis. P-values of the t-test were indicated in the table.

Columns are mean of triplicate experiments; bars, ±SD. * $p < 0.05$, ** $p < 0.01$, *** $p < 0.001$, **** $p < 0.0001$

Supplementary information 11

A. Combined NPB-PARPis treatment to PDO

Patient		Tissue type for PDO	Pathological subtype	Age at diagnosis (years)	FIGO stage	Surgery	Chemotherapy	metastasis	Family history
**PDO**	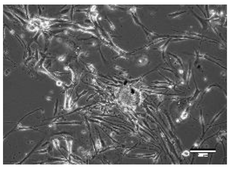	Endometrial carcinoma	Endometrioid carcinoma	53	FIGO II	yes	NY	Iliac lymph nodes	N

FIGO: The International Federation of Gynecology and Obstetrics. NY: Not yet; N:No

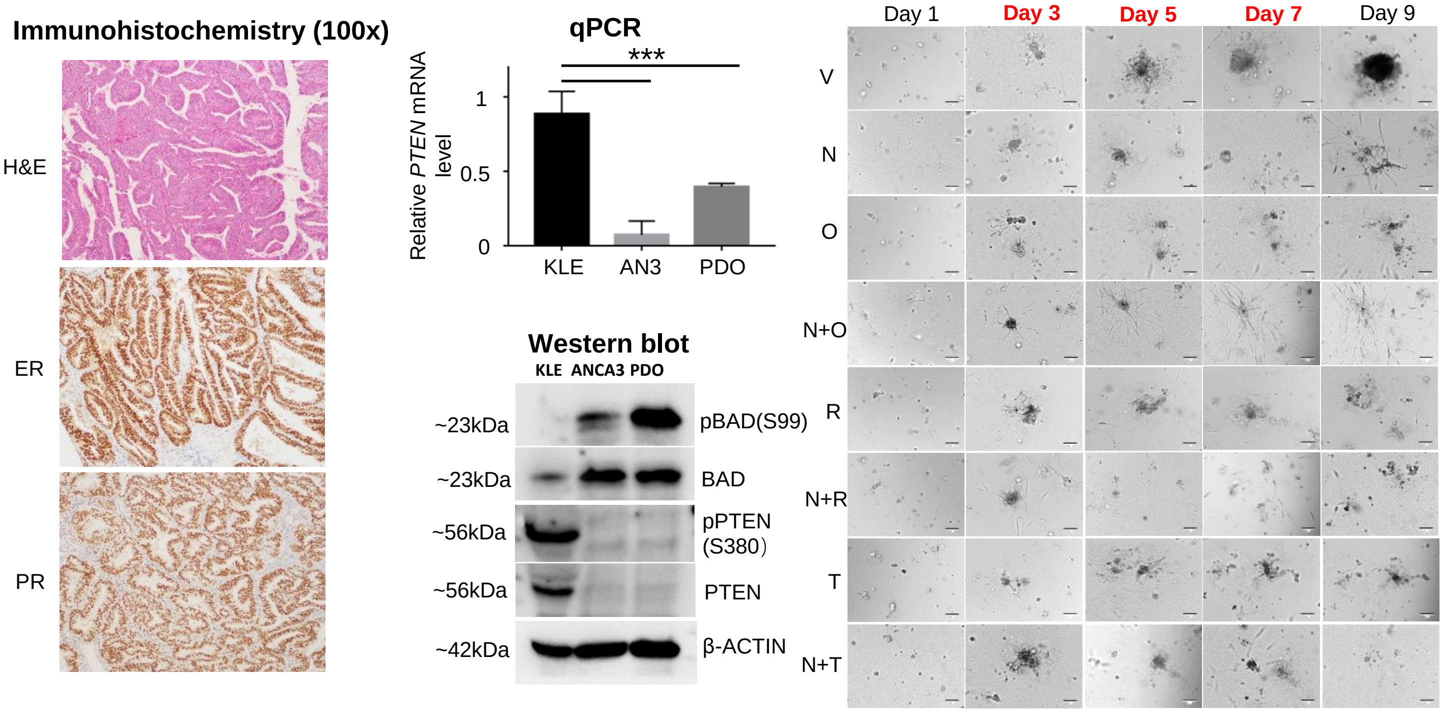

B. Scheme of xenograft protocol

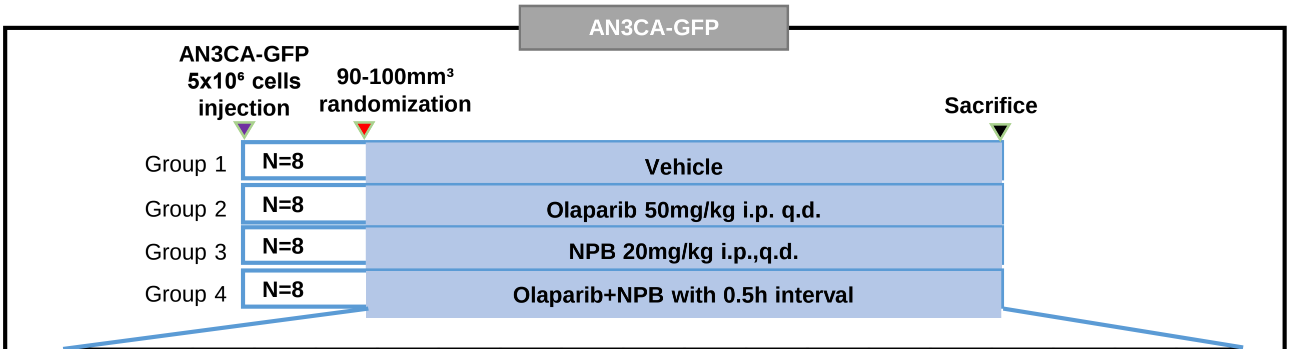

Day	1	2	3	4	5	6	7	8	9	10	11	12	13	14	15	16	17
Treat	+	+	+	+	+	+	+	+	+	+	+	+	+	+	+	+	+

C. AN3CA-EGFP cell

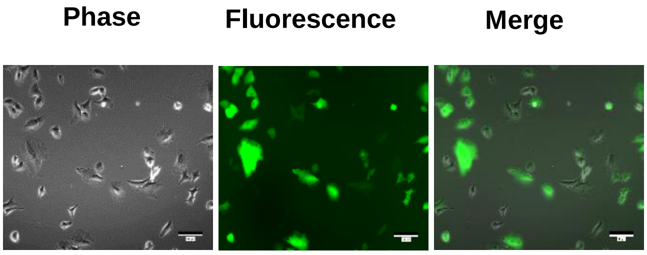

D. In vivo imaging

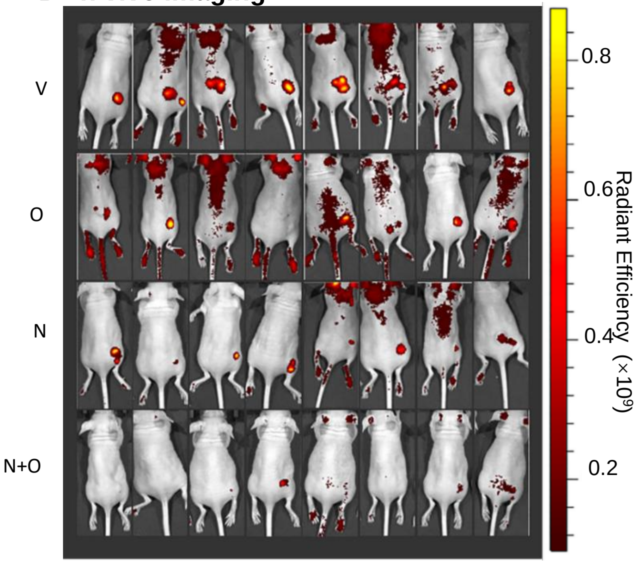

Supplementary information 11.

(A) Top: Clinical information of PDECO patient. Left below: Immunohistochemistry staining of hematoxylin and eosin (H&E), estrogen receptor (ER), and progestogen receptor (PR) in tumor tissue, magnification: 100x. Middle below: RT-qPCR and Western blot to confirm the PTEN level in the PDECO. Right below: Following 3 days post-embedding, pre-grown PDOs were treated with NPB (N), PARP inhibitors (Olaparib=O, Rucaparib=R, Talazoparib=T), or a combination every 2 days. Images of the PDOs were captured for the record every 2 days.

(B) Scheme for xenograft treatment: AN3CA-GFP cells were injected subcutaneously into the flank of the BALB/c athymic mice, respectively. In each cell line group, the mice were randomized into 4 indicated treatment groups (n = 8) and treated daily with vehicle, 20 mg/kg NPB, 50 mg/kg Olaparib, or a combination of both drugs for 17 days. Tumor volumes and mice weights were measured daily.

(C) Representative AN3CA-GFP fluorescent image.

(D) The xenografts in mice in each group were observed by a fluorescence imaging system (IVIS Spectrum, PerkinElmer, US) on the 18th day before sacrifice.

Supplementary information 12

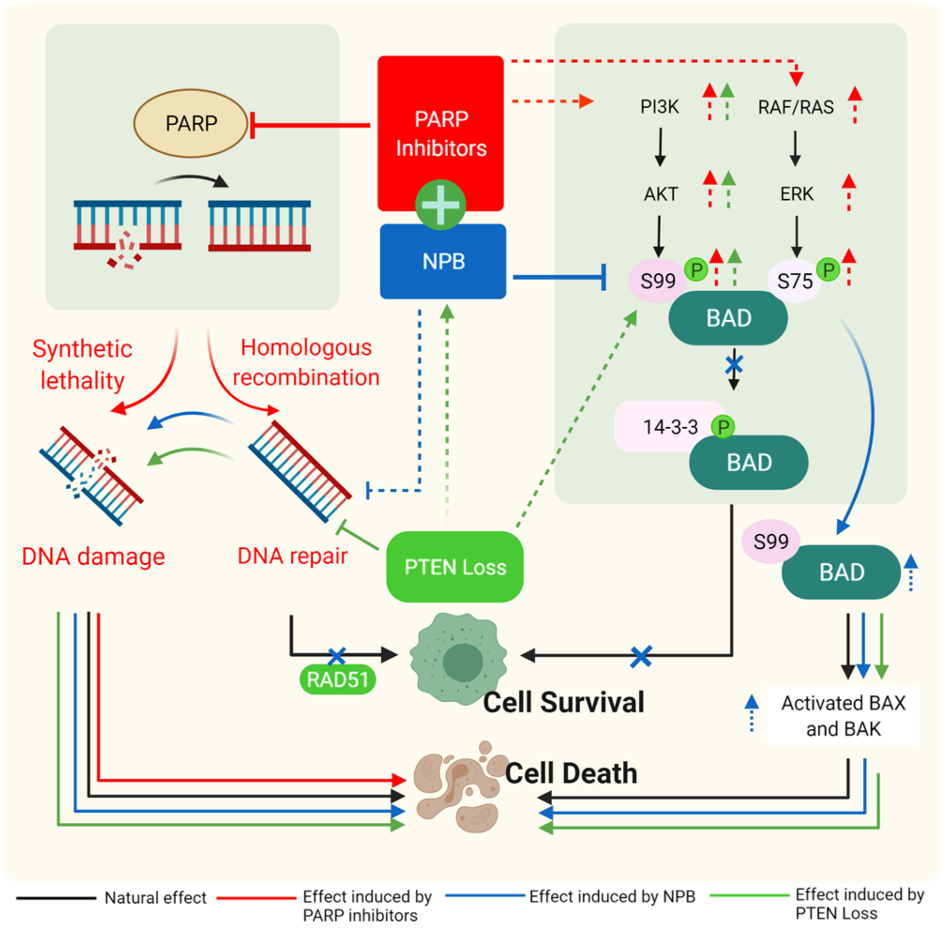

Supplementary Information 12.

Interaction between mitochondria-mediated apoptosis and the DNA damage process in *PTEN*-deficient EC provides a rational combination strategy of PARP inhibitors and NPB.

Poly ADP-Ribose polymerase (PARP), as the major responder of single-strand DNA break (SSB) repair through the mechanism of base excision repair (BER), is recruited to the site of DNA damage. PARP inhibitors (PARPis) cause apoptotic cell death through synthetic lethality in endometrial cancer (EC) cells deficient in genes involved in homologous recombination (HR), such as *PTEN*. Loss of *PTEN* impairs DNA double-strand break repair by blunting expression of the DNA repair protein RAD51 and hence is predictive of sensitivity to PARPis. *PTEN* deficiency is associated with activation of the PI3K-AKT-mTOR pathway which subsequently increases phospho-BAD(Ser99) levels. PARPis are also reported to induce activation of the PI3K/AKT/mTOR and RAS/MEK/ERK pathways. Both pathways converge at BCL-2–associated death promoter (BAD) protein, in which the RAS/MEK/ERK pathway and the pAKT70S6K of PI3K/AKT/mTOR pathway participate in BAD phosphorylation at Serine (S)75 and S99 respectively, leading to the formation of the 14-3-3/pBAD complex resulting in increased cell survival. Treatment of *PTEN*-deficient EC with BAD phosphorylation inhibitor NPB inhibits HR and promotes mitochondrial-mediated apoptosis. Interactions between mitochondrial-mediated apoptosis and the DNA damage/repair process in *PTEN*-deficient EC provide a mechanistic basis that a combination of a BAD phosphorylation inhibitor and a PARP inhibitor represents a potential therapeutic strategy for *PTEN*-deficient EC.

Supplementary information 13. Uncropped western blot images.

A. Uncropped western blot images for figure 3C.

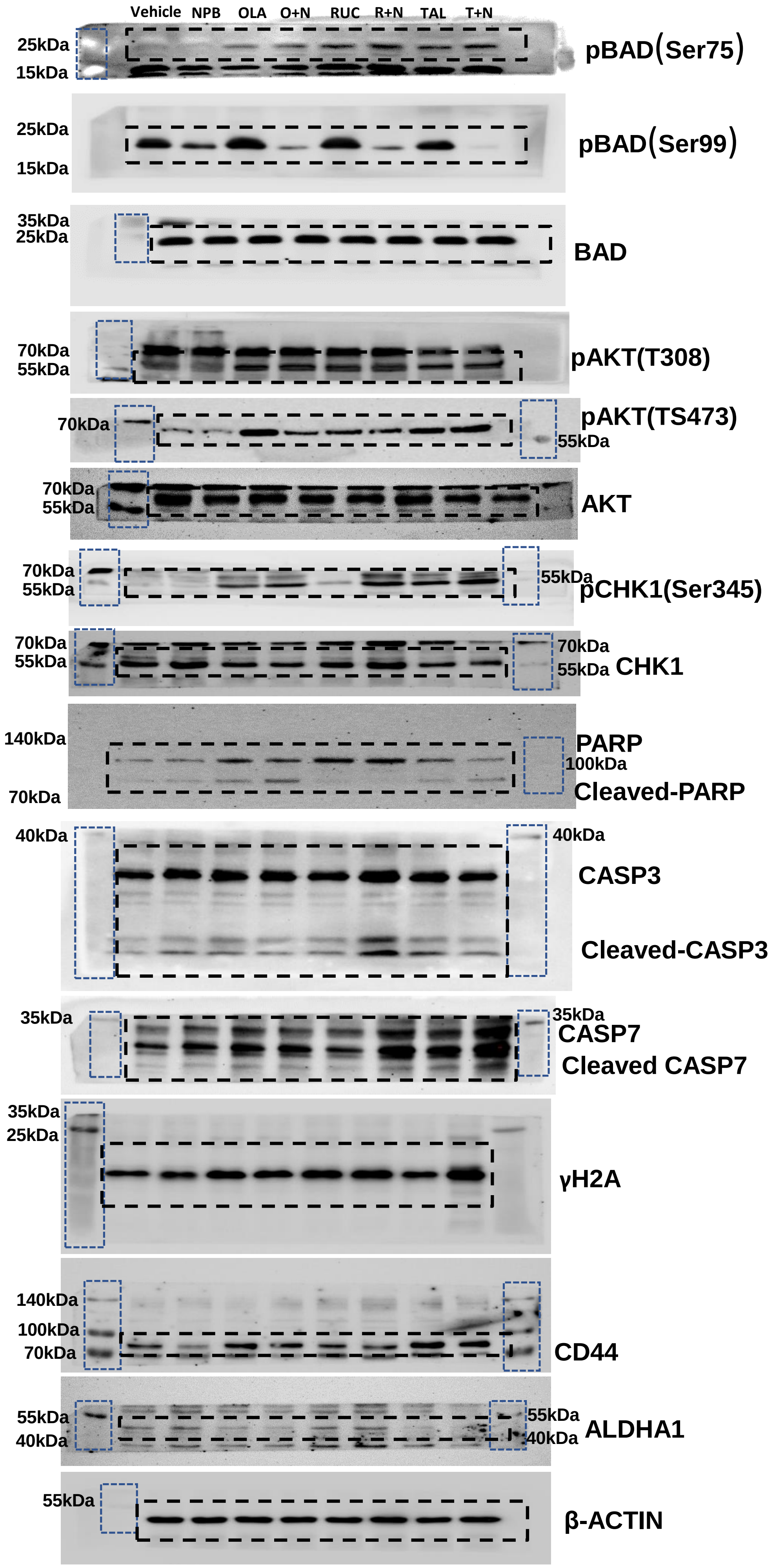

Supplementary information 13. Uncropped western blot images.

B. Uncropped western blot images for figure 4A.

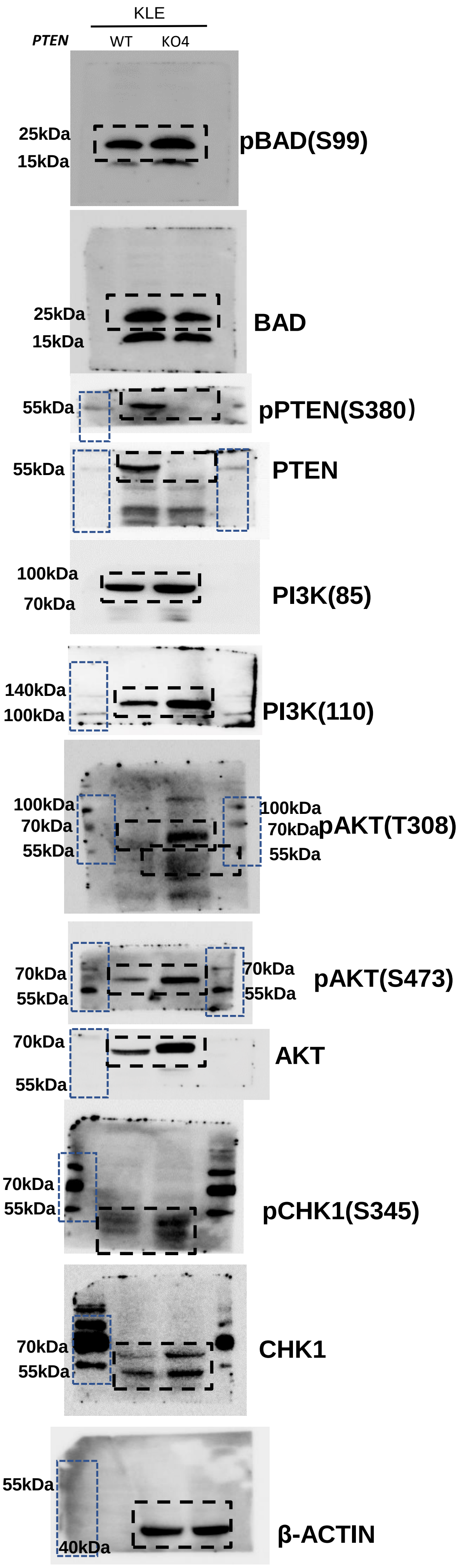

C. Uncropped western blot images for figure 4D.

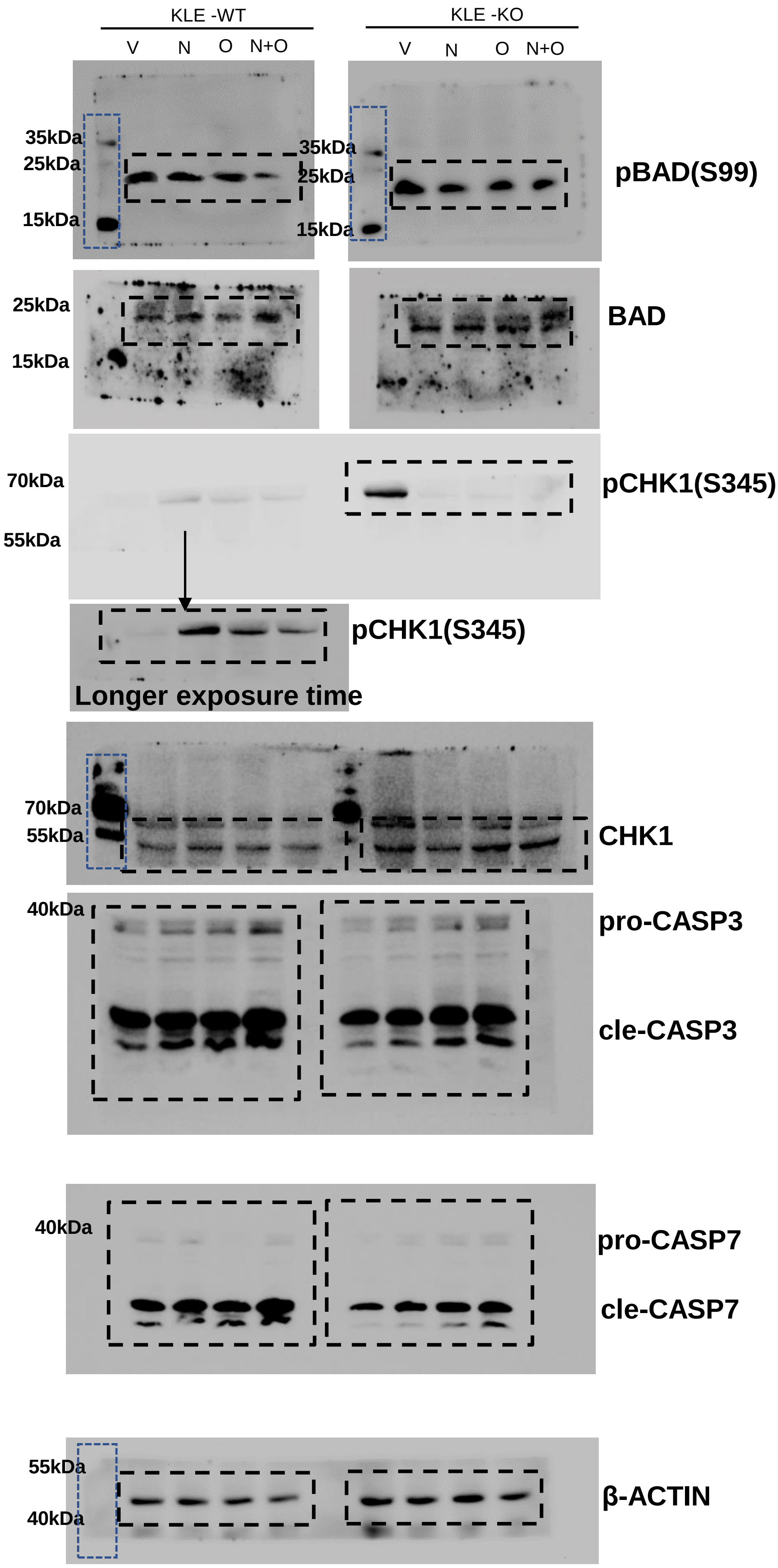

Supplementary information 13. Uncropped western blot images.

D. Uncropped western blot images for Supplementary information 3A

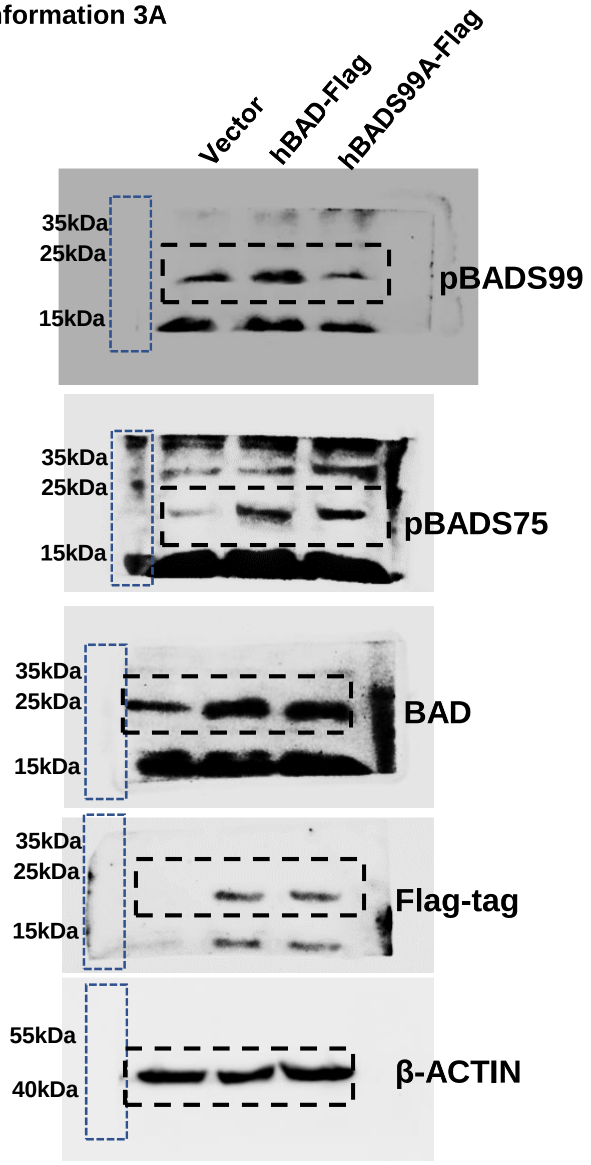

E. Uncropped western blot images for Supplementary information 3B

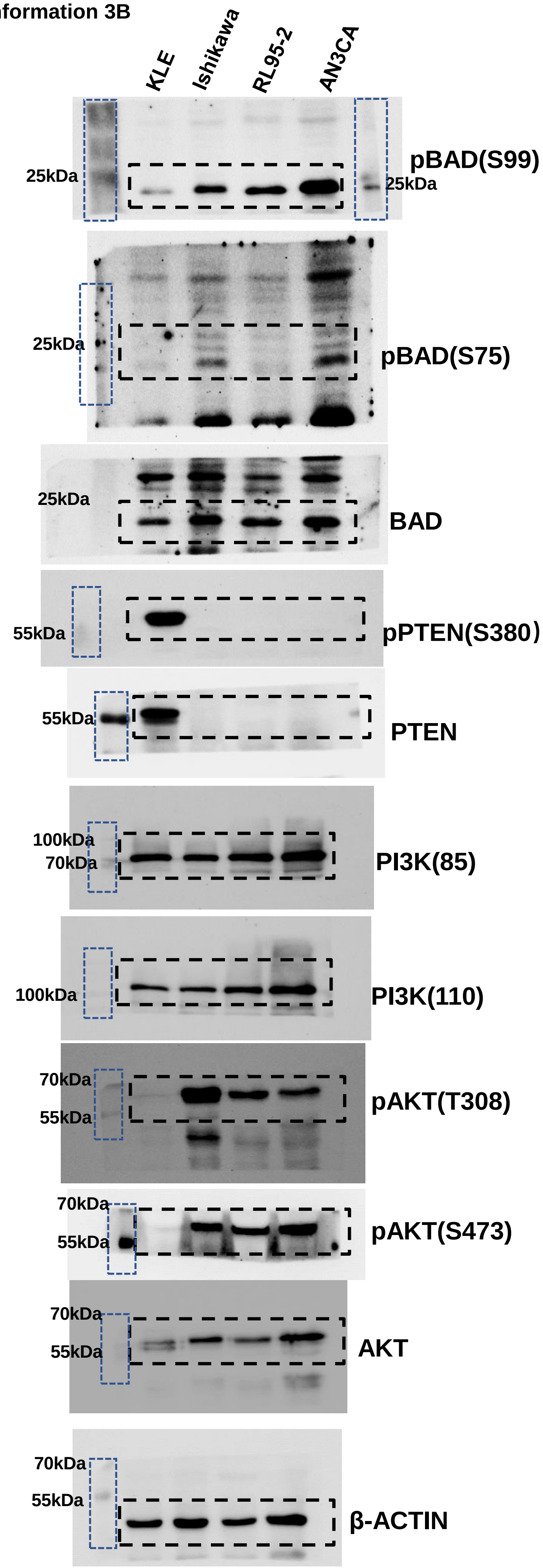

F. Uncropped western blot images for Supplementary information 9A

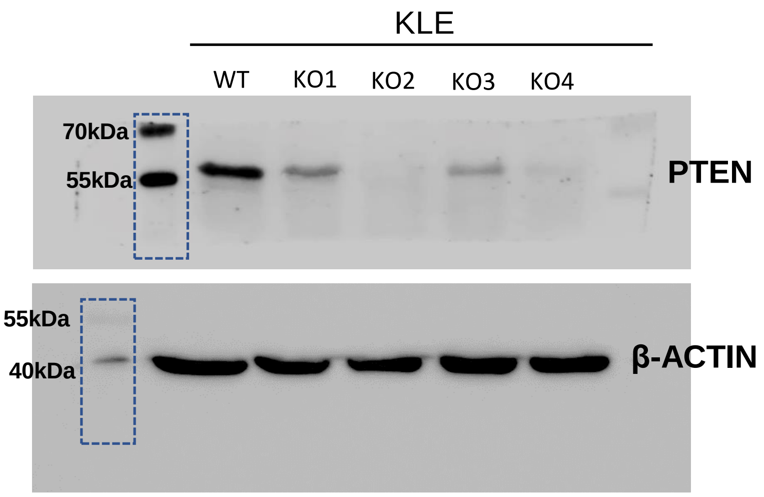

Supplementary information 13. Uncropped western blot images.

G. Uncropped western blot images for Supplementary information 11A

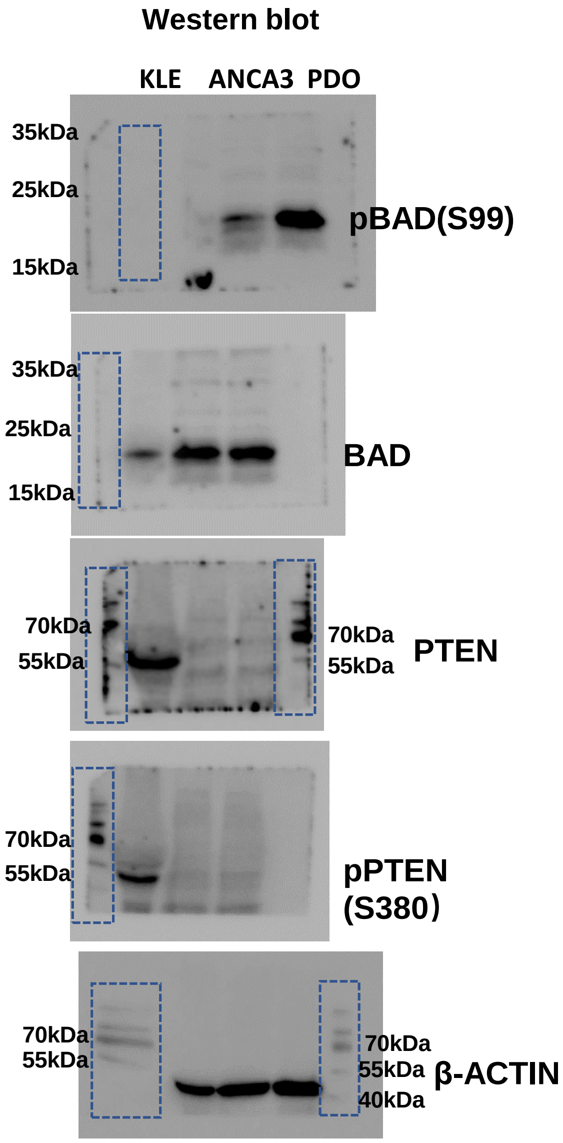

Supplementary information 13.

(A) Uncropped western blot images for figure 3C.

(B) Uncropped western blot images for figure 4A.

(C) Uncropped western blot images for figure 4D.

(D) Uncropped western blot images for Supplementary information 3A.

(E) Uncropped western blot images for Supplementary information 3B.

(F) Uncropped western blot images for Supplementary information 9A.

(G) Uncropped western blot images for Supplementary information 11A.

Supplementary References

1. Pandey V, *et al.* (2018) Discovery of a small-molecule inhibitor of specific serine residue BAD phosphorylation. *Proc Natl Acad Sci U S A* 115(44):E10505-E10514.
2. You M-L, *et al.* (2017) Trefoil factor 3 mediation of oncogenicity and chemoresistance in hepatocellular carcinoma is AKT-BCL-2 dependent. *Oncotarget* 8(24):39323.
3. Sulaiman NBS, *et al.* (2016) An azaspirane derivative suppresses growth and induces apoptosis of ER-positive and ER-negative breast cancer cells through the modulation of JAK2/STAT3 signaling pathway. *International journal of oncology* 49(3):1221-1229.
4. Wang Y, *et al.* (2020) Pharmacological Inhibition of BAD Ser99 Phosphorylation Enhances the Efficacy of Cisplatin in Ovarian Cancer by Inhibition of Cancer Stem Cell-like Behavior. *ACS Pharmacology & Translational Science*.
5. Maenhoudt N, *et al.* (2020) Developing Organoids from Ovarian Cancer as Experimental and Preclinical Models. *Stem Cell Reports* 14(4):717-729.
6. Kopper O, *et al.* (2019) An organoid platform for ovarian cancer captures intra- and interpatient heterogeneity. *Nat Med* 25(5):838-849.
7. Boehnke K, *et al.* (2016) Assay Establishment and Validation of a High-Throughput Screening Platform for Three-Dimensional Patient-Derived Colon Cancer Organoid Cultures. *J Biomol Screen* 21(9):931-941.
8. Zhao H, *et al.* (2019) Sphereforming assay vs. organoid culture: Determining longterm stemness and the chemoresistant capacity of primary colorectal cancer cells. *Int J Oncol* 54(3):893-904.
9. Berg HF, *et al.* (2021) Patient-derived organoids reflect the genetic profile of endometrial tumors and predict patient prognosis. *Communications Medicine* 1(1):20.
